# Riding the wave of genomics to investigate aquatic coliphage diversity and activity

**DOI:** 10.1111/1462-2920.14590

**Published:** 2019-04-04

**Authors:** Slawomir Michniewski, Tamsin Redgwell, Aurelija Grigonyte, Branko Rihtman, Maria Aguilo‐Ferretjans, Joseph Christie‐Oleza, Eleanor Jameson, David J. Scanlan, Andrew D. Millard

**Affiliations:** ^1^ School of Life Sciences University of Warwick Gibbet Hill Road, Coventry CV4 7AL UK; ^2^ Department of Genetics and Genome Biology University of Leicester, University Road Leicester LE1 7RH UK

## Abstract

Bacteriophages infecting Escherichia coli (coliphages) have been used as a proxy for faecal matter and water quality from a variety of environments. However, the diversity of coliphages that is present in seawater remains largely unknown, with previous studies largely focusing on morphological diversity. Here, we isolated and characterized coliphages from three coastal locations in the United Kingdom and Poland. Comparative genomics and phylogenetic analysis of phage isolates facilitated the identification of putative new species within the genera *Rb69virus* and *T5virus* and a putative new genus within the subfamily *Tunavirinae*. Furthermore, genomic and proteomic analysis combined with host range analysis allowed the identification of a putative tail fibre that is likely responsible for the observed differences in host range of phages vB_Eco_mar003J3 and vB_Eco_mar004NP2.

## Introduction

Bacteriophages are a key component of microbial communities playing important roles such as increasing the virulence and driving the evolution of their bacterial hosts and influencing major biogeochemical cycles (see Breitbart *et al.,* 2007, 2018; Suttle, 2007; Perez Sepulveda *et al.,* 2016 for reviews). It is estimated that there are 10^31^ viruses in the biosphere with each millilitre of seawater containing millions of these viruses (Suttle, 2017), largely infecting the numerically dominant bacterial genera *Synechococcus*, *Prochlorococcus* and SAR11 (Suttle and Chan, 1993; Wilson *et al.,* 1993; Sullivan *et al.,* 2003; Mühling *et al.,* 2005; Kang *et al.,* 2013; Zhao *et al.,* 2013; Deng *et al.,* 2014). Culture‐ and metagenomics‐based approaches have shed much light on their genetic diversity (Millard *et al.,* 2009; Sullivan *et al.,* 2010; Hurwitz *et al.,* 2013; Brum *et al.,* 2015; Gregory *et al.,* 2016) including the description of several previously unknown phage groups that are widespread in the environment (Sabehi *et al.,* 2012; Holmfeldt *et al.,* 2013; Kang *et al.,* 2013; Zhao *et al.,* 2013; Chan *et al.,* 2015).

In the context of marine systems, bacteriophage infecting *Escherichia coli*, commonly referred to as coliphage, have perhaps received less attention even though they have been widely studied as a proxy for drinking water quality and the presence of faecal coliforms and enteric viruses (Hilton and Stotzky, 1973; Vaughn and Metcalf, 1975; Snowdon and Coliver, 1989; Palmateer *et al.,* 1991). Thus, much is known about how the use of different *E. coli* strains or growth media used can lead to variable estimates of phage abundance (Havelaar and Hogeboom, 1983; Jofre, 2009; Muniesa *et al.,* 2013) and this has resulted in global standards for using coliphages as a measure of water quality (ISO, 2016). These standards rely on the use of *E. coli* C strains derived from ATCC13706, which have been shown to detect increased titres over *E. coli* B and *E. coli* K12 derivatives (Havelaar and Hogeboom, 1983). The presence of coliphage in marine waters is assumed to be the result of anthropogenic input and not due to any ongoing increase *in situ* as a result of infection and replication (Borrego *et al.,* 1990). However, while the consensus seems to be that coliphage replication *in situ* is not a significant issue (Jofre, 2009), more recent research provides evidence that coliphages may well replicate in the environment (Reyes and Jiang, 2010).

Regarding the diversity of coliphages found in seawater, studies have largely focused on morphological diversity (Muniesa *et al.,* 1999; Reyes and Jiang, 2010; Burbano‐Rosero *et al*., 2011; Jofre *et al.,* 2016) and assessing the number and range of *E. coli* hosts they can infect. This has shown that many coliphages have a broad host range, with detection of coliphages comprising members of the *Siphoviridae* and *Myoviridae* families off the Californian (Reyes and Jiang, 2010) and Brazilian coasts (Burbano‐Rosero *et al*., 2011) with *Siphoviridae* being the most frequently observed taxa.

Coliphages in general are one of the most sequenced phage types with ~450 complete phage genomes within Genbank, isolated from a variety of sources including animal faeces (Niu *et al.,* 2014; Smith *et al.,* 2015; Sazinas *et al.,* 2016; Golomidova *et al.,* 2018), human faeces (Dalmasso *et al.,* 2016), urine (Malki *et al.,* 2016), river water (Alijošius *et al.,* 2017), agricultural surface waters (Liao *et al.,* 2018), lagoons (Ngazoa‐Kakou *et al.,* 2018), sewage (Trotereau *et al.,* 2017) and animal slurries (Sazinas *et al.,* 2016). However, much less is known about the genetic diversity of coliphages in seawater. To shed light on this, we isolated coliphages from three locations in the United Kingdom and Poland and undertook genomic and proteomic characterization of the isolated phages, to provide insights into their phylogenetic position and functional potential.

## Results

### *Newly isolated coliphages—phylogeny and taxonomy*

For all samples tested, the titre of coliphage detected was extremely low, generally <1 pfu ml^−1^ (Table 1). A total of 10 phages were isolated and purified from three different seawater samples and one phage from a freshwater urban pond. These phage were purified and their genomes sequenced to assess their genomic diversity (Table 1). Coliphage genomes were first compared against each other using MASH (Ondov *et al.,* 2016) in an all‐versus‐all approach, which revealed three groups of phages based on similarity to each other: Group1: vB_Eco_mar003J3 and vB_Eco_mar004NP2; Group2: vB_Eco_mar005P1, vB_Eco_mar006P2, vB_Eco_mar007P3 vB_Eco_mar008P4 and vB_Eco_mar009P5; Group3: vB_Eco_swan01, vB_Eco_mar001J1 and vB_Eco_mar002J2. Each phage was then compared against a database of all complete phage genomes using MASH (April 2018) (Ondov *et al.,* 2016).

**Table 1:** Locations of water samples, titre of coliphages detected and phage isolates from each location. ND—titre not determined.

Water source	Titre	Phage isolates	Date of isolation
Oliva stream estuary, Jelitkowo, Gdansk, Poland	0.28 pfu ml^−1^	vB_Eco_mar001J1	30.01.2017
		vB_Eco_mar002J2	30.01.2017
		vB_Eco_mar003J3	30.01.2017
Martwa Wisla Estuary, Nowy Port, Gdansk, Poland	0.11 pfu ml^−1^	vB_Eco_mar004NP2	30.01.2017
Swanswell Pool, Coventry, United Kingdom	0.0125 pfu ml^−1^	vB_Eco_swan01	08.12.2016
Great Yarmouth, United Kingdom	ND	vB_Eco_mar005P1	08.12.2016
		vB_Eco_mar006P2	08.12.2016
		vB_Eco_mar007P3	08.12.2016
		vB_Eco_mar008P4	08.12.2016
		vB_Eco_mar009P5	08.12.2016

### *Genus* Rb69virus

Phages vB_Eco_mar005P1, vB_Eco_mar006P2, vB_Eco_mar007P3, vB_Eco_mar008P4 and vB_Eco_mar009P5 had greatest mash similarity to phages APCEc01 (accession KR422352) and *E. coli* O157 typing phage 3 (accession KP869101), neither of which are currently classified by the ICTV but are similar to other phages within the *Tevenvirinae*. To further investigate the phylogeny of these phages, the gene encoding the major capsid protein (*g23*) was used to construct a phylogeny, as it is widely used as a phylogenetic marker including being used previously to classify phages within the *Tevenvirinae* (Adriaenssens and Cowan, 2014). The *g23* sequence for the five newly isolated phages (vB_Eco_mar005P1, vB_Eco_mar006P2, vB_Eco_mar007P3, vB_Eco_mar008P4 and vB_Eco_mar009P5) were identical, therefore only one copy was included in the phylogenetic analysis. The analysis placed the new phage isolates within a clade that contains APCEc01, *E. coli* O157 typing phage 3, HX01, vB_EcoM_JS09 and RB69 (Supporting Information Fig. S1). The latter three of these form part of the genus *Rb69virus,* suggesting the newly isolated phages are also part of this genus (Supporting Information Fig. S1).

**Figure 1:**
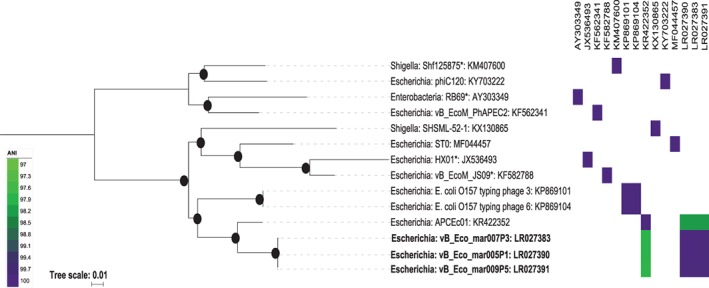
Phylogenetic analysis of phages within the genus *Rb69virus*. The tree is based on the nucleotide sequence of nine concatenated genes [*nrdC* (GeneID:1494209), *rnlA* (GeneID:1494352), *ndd* (GeneID:1494384), *regA* (GeneID:1494173), *g52* (GeneID:1494381), *g14* (GeneID:1494292), td (GeneID:1494357), *g053* (GeneID:1494168) and *g30.3* (GeneID:1494331)] using a GTR+F+ASC+R2 model of evolution, with 1000 bootstrap replicates using IQTREE (Nguyen *et al.,* 2015). Current phage species as defined by the ICTV are marked with an *. Bootstrap values above 70% are marked with a filled circle, with the size proportional to the bootstrap value. The ANI value between phages is represented as a heatmap, with only values >97% coloured. The phages included in the tree are RB69 (acc:AY303349), Shf125875 (acc: KM407600), phiC120 (acc:KY703222), vB_EcoM_PhAPEC2 (acc:KF562341), SHSML‐52‐1 (acc:KX130865), ST0 (acc:MF044457), HX01 (acc:JX536493), vB_EcoM_JS09 (acc:KF582788), E. coli O157 typing phage 3 (acc:KP869101), E. coli O157 typing phage 6 (acc:KP869104), APCEc01 (acc:KR422352), and vB_Eco_mar005P1 (acc:LR027390). [Color figure can be viewed at wileyonlinelibrary.com]

The genomes of phages from the genus *Rb69virus* were further compared together with phage phiE142, which is classified as part of the *Rb69virus* genus, and has an ANI of ~91% compared to the new isolates in this study. The ANI of all phages was calculated and compared in an all‐v‐all comparison. The newly isolated phages possessed an ANI of >95% compared to HX01, JS09 and RB69 suggesting they are representatives of one of these species based on current standards (Adriaenssens and Brister, 2017). In fact, with the exception of phiE142 (Supporting Information Table S1), all phages had an ANI >95% with at least one other phage (Fig. 1, Supporting Information Table S1). To further elucidate the evolutionary history of these phages, a core gene analysis was carried out. In the process of doing this, it became apparent phiE142 was ~50 kb smaller than the other phages within this group. Furthermore, it lacks essential genes that encode the major structural proteins and small and large subunit terminase. Therefore, it was excluded from further analysis as it is incomplete despite being described as complete (Amarillas *et al.,* 2016).

The core‐genome of the genus *Rb69virus* consisted of 170 genes, which accounted for 60.3%‐68.3% of the total genes in each phage (Supporting Information Table S1). To further classify these phages, the GET_PHYLOMARKERS pipeline was used to identify suitable genes for phylogenetic analysis (Vinuesa *et al.,* 2018). Only 89 genes were identified that did not show signs of recombination when tested with Phi test (Bruen, 2005). This test was carried out as recombination is known to result in inaccurate phylogenies and branch lengths (Didelot and Maiden, 2010). Eighty‐six of these passed further filtering to remove genes that were considered significant outliers using the KDETREES test (Weyenberg *et al.,* 2014). The resulting top nine genes (Supporting Information Table S1) as determined via GET_PHYLOMARKERS (Vinuesa *et al.,* 2018) were selected for phylogenetic analysis, and a concatenated alignment was used for phylogenetic analysis. Phylogenetic analysis placed the newly isolated phages in a clade with *Escherichia* phage APCEc01 (accession: KR422352) further confirming they are part of the genus *Rb69virus*.

Current taxonomy classifies RB69, HX01, JS09 and Shf125875 as four species within the genus *Rb69virus* (Kropinski *et al.,* 2015a). This is based on the definition that phage species with >95% similarity based on BLASTn to another phage are the same species (Adriaenssens and Brister, 2017). In our analysis, the nucleotide identity between genomes was estimated using ANI by fragmentation of the genomes (Goris *et al.,* 2007) rather than simple BLASTn comparison (Fig. 1). Using an ANI value of >95% did not differentiate between phage species and maintained the current taxonomy, with each phage having an ANI >95% to multiple phages suggesting that *Rb69virus* should contain only two species. Nevertheless, the phylogeny clearly supports multiple species within the *Rb69virus* genus, suggesting a cut‐off of 95% ANI may not be suitable (Fig. 1). Consequently, if an ANI of >97% was used to differentiate species, this closely resembled the observed phylogeny (Fig. 1). The higher ANI cut‐off value discriminates between RB69 and Shf125875, maintaining their previous classification as separate species. Furthermore, this will split the genus *Rb69virus* into ten species, which are represented by Shf125875, phiC120, RB69, vB_EcoM_PhAPEC2, SHSML‐52‐1, STO, HX01, JS09, *E. coli* O157 typing phage 3 (strains *E.coli* O157 typing phage 6) and APCEc01 (including the five new isolates in this study). This suggests the five phage isolates identified in this study are representatives of a new species within the genus *Rb69virus* (order *Caudovirales*, family *Myoviridae*, subfamily *Tevenvirinae)*.

The phage isolated in this study vB_Eco_mar005P1, vB_Eco_mar006P2 and vB_ Eco_mar008P4 are identical. Phages vB_Eco_mar007P3 and vB_Eco_mar009P5 share the same gene content but are distinguishable by differences in single nucleotide variations.

### *Genus* T5virus

A similar approach was used for classification of the newly isolated phages vB_Eco_mar003J3 and vB_Eco_mar004NP2, which were most similar to phages within the genus *T5virus* based on MASH identity. All phages that are currently listed as part of the genus *T5virus* were extracted from GenBank (April 2018). Initially, the gene encoding DNA polymerase was used to construct a phylogeny, which has previously been used for the classification of phages within the genus *T5virus* (Sváb *et al.,* 2018) (Supporting Information Table S2). This confirmed that phages vB_Eco_mar003J3 and vB_Eco_mar004NP2 were related to other phages within the genus *T5virus* (Supporting Information Fig. S2). Determination of the core‐genome revealed 19 genes formed the core when using 90% identity for identification of orthologues using ROARY. However, when using this value and then applying the same filtering parameters as used for the genus *Rb69virus*, no genes were deemed suitable for phylogenetic analysis. Therefore, an iterative process was used whereby the identity between proteins was lowered by 5% on each run of ROARY and the analysis repeated until a number of phylogenetic markers passed the filtering criteria. This was reached at a protein identity of 75%. At this point, 44 core genes were identified, of which only 14 passed further filtering steps (Supporting Information Table S2). The top nine markers as selected by the GET_PHYLOMARKERS pipeline were used for phylogenetic analysis (Vinuesa *et al.,* 2018).

**Figure 2:**
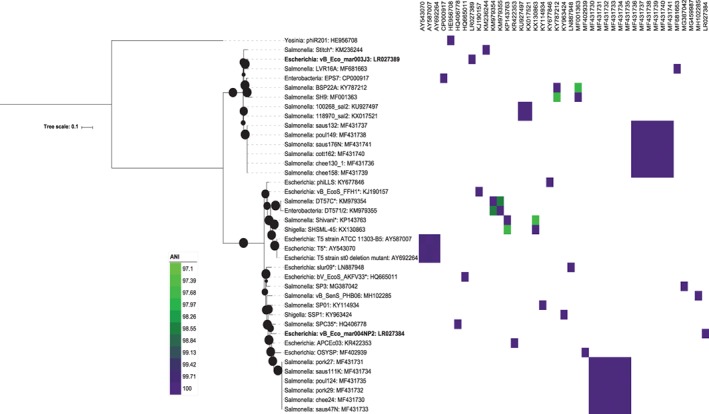
Phylogenetic analysis of phages within the genus *T5virus*. The tree is based on the nucleotide sequence of two concatenated genes (locus tags: MAR004NP2_00031 and MAR004NP2_00005) using a GTR+F+ASC+R2 model of evolution, with 1000 bootstrap replicates using IQTREE (Nguyen *et al.,* 2015). Current phage species as defined by the ICTV are marked with an *. Bootstrap values above 70% are marked with a filled circle, with the size proportional to the bootstrap value. The ANI value between phages is represented as a heatmap, with only values >97% coloured. The phages included in the tree are T5 (acc:AY543070), T5 strain ATCC 11303‐B5 (acc:AY587007), T5 strain st0 deletion mutant (acc:AY692264), EPS7 (acc:CP000917), phiR201 (acc:HE956708), SPC35 (acc:HQ406778), bV_EcoS_AKFV33 (acc:HQ665011), AvB_EcoS_FFH1 (acc:KJ190157), Stitch (acc:KM236244), DT57C (acc:KM979354), DT571/2 (acc:KM979355), Shivani (acc:KP143763), APCEc03 (acc:KR422353), 100268_sal2 (acc:KU927497), 118970_sal2 (acc:KX017521), SP01 (acc:KY114934), phiLLS (acc:KY677846), BSP22A (acc:KY787212), SSP1 (acc:KY963424), slur09 (acc:LN887948), SH9 partial (acc:MF001363), OSYSP (acc:MF402939), chee24 (acc:MF431730), pork27 (acc:MF431731), pork29 (acc:MF431732), saus47N (acc:MF431733), saus111K (acc:MF431734), poul124 (acc:MF431735), chee130_1 (acc:MF431736), saus132 (acc:MF431737), poul149 (acc:MF431738), chee158 (acc:MF431739), cott162 (acc:MF431740), saus176N (acc:MF431741), LVR16A partial (acc:MF681663), SP3 partial (acc:MG387042), vB_SenS_PHB06 (acc:MH102285), vB_Eco_mar003J3 (acc:LR027389) and vB_Eco_mar004NP2 (acc:LR027384). [Color figure can be viewed at wileyonlinelibrary.com]

Phylogenetic analysis on the selected marker genes confirmed that vB_Eco_mar004NP2 and vB_Eco_mar003J3 fall within the genus *T5virus* (order *Caudovirales*, family *Siphoviridae)* (Fig. 2). Phage vB_Eco_mar004NP2 is a sister clade to that of phage SPC35 (HQ406778) and vB_Eco_mar003J3 and a sister group to that of phage LVR16A (MF681663) (Fig. 2). Phage vB_Eco_mar004NP2 represents a new species within the genus *T5virus* as it has <95% ANI with any other phage within the genus (Adriaenssens and Brister, 2017). For phage vB_Eco_mar003J3, it is not clear if the phage represents a new species. It has an ANI >95% with phages saus132 and paul149, which have recently been described as new species (Sváb *et al.,* 2018). However, these phages are not the closest group based on a phylogenetic analysis (Fig. 2). When an ANI value of >97% is used then currently defined species are more congruent with the observed phylogenetic analysis, suggesting vB_Eco_mar003J3 is a novel species (Fig. 2). Applying this threshold of 97% ANI across the entire genus would maintain the current species and create a total of 23 species across the genus.

### *Tunavirinae*

Phages vB_Eco_mar001J1, vB_Eco_mar002J2 and vB_Eco_swan01 had greatest nucleotide sequence similarity to pSf‐1 and SECphi27, which are the members of the subfamily *Tunavirinae*. Phage isolates vB_Eco_mar001J1 and vB_Eco_mar002J2 were found to be identical.

To classify the newly isolated phages, a phylogenetic analysis was carried out using the gene encoding the large subunit terminase that has previously been used to classify phages within the subfamily *Tunavirinae* by the ICTV (Kropinski *et al.,* 2015b). The analysis included all current members of the subfamily *Tunavirinae* (April 2018). The newly isolated phages vB_Eco_mar001J1, vB_Eco_mar002J2 and vB_Eco_swan01 form a clade with phages pSf‐1, SECphi27 and Esp2949‐1 (Supporting Information Fig. S3). This clade is a sister to the clades that represent the previously defined genera *KP36virus* and *TLSvirus*, thus clearly placing these new phages within the subfamily *Tunavirinae* (order *Caudovirale*s, family *Siphoviridae*) (Supporting Information Fig. S3).

**Figure 3:**
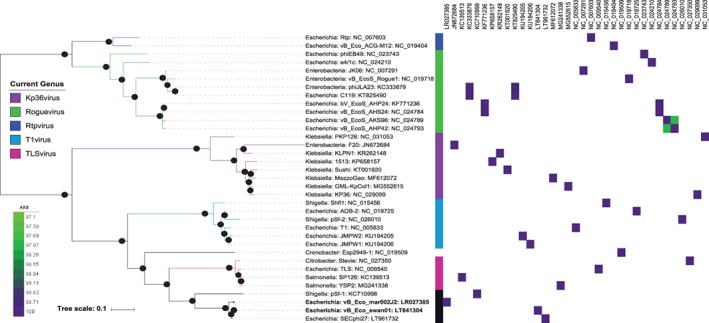
Phylogenetic analysis of phages within the subfamily *Tunavirinae*. The tree is based on the nucleotide sequence of four concatenated genes (locus tags, MAR001J1_00001, MAR001J1_00004, MAR001J1_00010, and MAR001J1_00077) using a GTR+F+ASC+G4 model of evolution, with 1000 bootstrap replicates using IQTREE (Nguyen *et al.,* 2015). Current phage genera as defined by the ICTV are marked with the first coloured strip chart. Bootstrap values above 70% are marked with a filled circle, with the size proportional to the bootstrap value. The ANI value between phages is represented as a heatmap, with only values >97% coloured. The phages included in the tree are Rtp (acc:NC_007603), vB_Eco_ACG‐M12 (acc:NC_019404), phiEB49 (acc:NC_023743), e4/1c (acc:NC_024210), JK06 (acc:NC_007291), vB_EcoS_Rogue1 (acc:NC_019718), phiJLA23 (acc:KC333879), C119 (acc:KT825490), bV_EcoS_AHP24 (acc:KF771236), vB_EcoS_AHS24 (acc:NC_024784), vB_EcoS_AKS96 (acc:NC_024789), vB_EcoS_AHP42 (acc:NC_024793), PKP126 (acc:NC_031053), F20 (acc:JN672684), KLPN1 (acc:KR262148), 1513 (acc:KP658157), Sushi (acc:KT001920), MezzoGao (acc:MF612072), GML‐KpCol1 (acc:MG552615), KP36 (acc:NC_029099), Shfl1 (acc:NC_015456), ADB‐2 (acc:NC_019725), pSf‐2 (acc:NC_026010), T1 (acc:NC_005833), JMPW2 (acc:KU194205), JMPW1 (acc:KU194206), Esp2949‐1 (acc:NC_019509), Stevie (acc:NC_027350), TLS (acc:NC_009540), SP126 (acc:KC139513), YSP2 (acc:MG241338), pSf‐1 (acc:KC710998), vB_Eco_swan01 (acc:LT841304), SECphi27 (acc:LT961732) and vB_Eco_mar002J2 (acc:LR027385). [Color figure can be viewed at wileyonlinelibrary.com]

To further clarify the phylogeny of these phages, a core gene analysis of all members of the subfamily *Tunavirinae* was carried out. Given these phage form part of a taxonomic sub‐family, using ROARY with similarity cut‐off values of 90% resulted, unsurprisingly, in the detection of no core genes. Therefore, an alternative method was used using an orthoMCL approach from within the GET_HOMOLOGUES software (Contreras‐Moreira and Vinuesa, 2013). OrthoMCL‐based analysis identified a core of only nine genes, which were then filtered in the same manner as for the *Rb69virus* and *T5virus* genera. A phylogeny was then constructed based on the concatenated alignment of four core genes (Fig. 3). Phylogenetic analysis confirmed the previously defined genera within *Tunavirinae*, with the five genera of *Kp36virus*, *Roguevirus*, *Rtpvirus*, *T1virus* and *TLSvirus* also supported by good bootstrap support values (Fig. 3). Furthermore, a clade which is sister to that of the genus *TLSvirus* was identified with good bootstrap support comprising vB_Eco_mar001J1, vB_Eco_mar002J2, vB_Eco_swan01, SECphi27 (accession KC710998) and pSf‐1 (accession NC_021331). Their clear separation from existing genera within the subfamily suggests this clade is a new genus. The phages within this putative genus all share an ANI >75% with other phages in the genus, compared to 60%–70% ANI with phages in the other described genera within the *Tunavirinae*. All phages within the putative genus have a conserved genome organization and share thirty orthologues. We propose that this clade represents a new genus and should be named as *pSFunavirus* after pSF‐1, the first representative isolate (Woo *et al.,* 2013). Furthermore, we propose the unclassified phage Esp2949‐1 (NC_019509) is the sole representative of a new genus, as it does not currently fit within currently defined genera. Phylogenetic analysis indicates that phages of the genus *TL1virus*, *TLSvirus*, *psFunavirus* all have a common ancestor, with Esp2949‐1 ancestral to phages in the genus *TL1virus* and *psFunavirus*. (Fig. 3). Comparative genomic analysis also supports this, with Esp2949‐1 having <70% ANI to phages of the genera *TL1virus* or *TLSvirus,* its closest relatives. Phages within the putative genus *psFunavirus* were further analysed to determine the number of species. Using a cut‐off of 95% or 97% ANI, the genus will contain three species vB_Eco_swan01 (SECphi27 and vB_Eco_swan01), vB_Eco_mar002J2 (vB_Eco_mar001J1 and vB_Eco_mar002J2 which are identical) and the orphan species pSF‐1.

Phylogenetic analysis demonstrated that of the 10 phages isolated, five represented novel species. A representative of each of these newly identified groups was further characterized both morphologically and physiologically. The representative phages were vB_Eco_swan01 and vB_Eco_mar002J2 (new species within the *Tunavirinae*), vB_Eco_mar003J3 and vB_Eco_mar004NP2 (new species within *T5virus*), and vB_Eco_mar005P1 (new species within *Rb69virus*).

### *Genomic properties*

**Figure 4:**
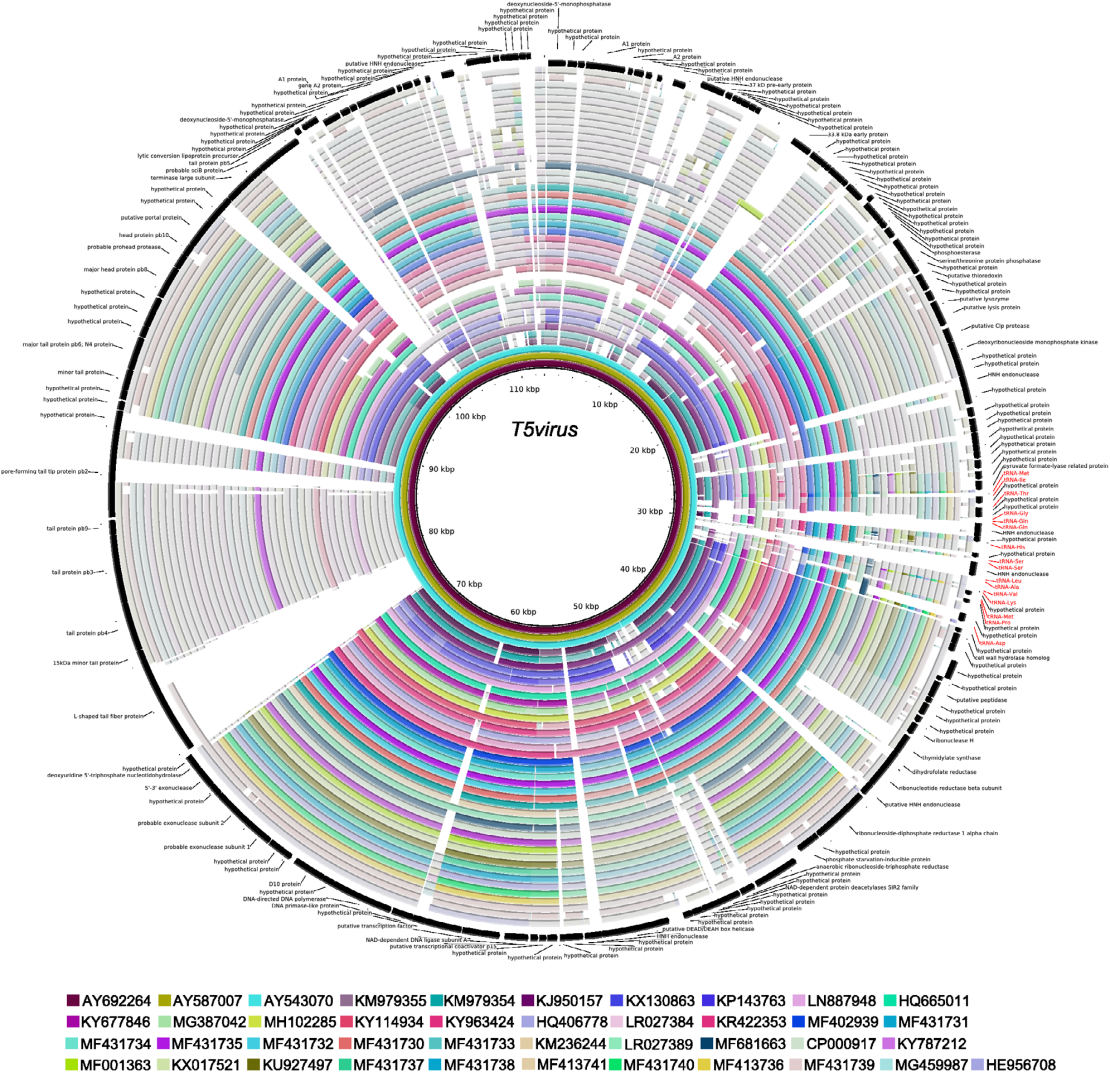
Genomic comparison of phages within the genus *T5virus*. All phages were compared to phage T5 (accession: AY692264) with BRIG (Alikhan *et al.,* 2011) using blastn settings of minimum e‐value 0.001 and minimum length of 100. Each phage is represented by a single ring of different colour. Nucleotide identity of 80%–100% is shaded in colour for each ring, with darker shading representing higher identity. An identity between 50%–80% is shaded in light grey. The outer two rings contain the genes from T5 (accession: AY692264) and annotation. [Color figure can be viewed at wileyonlinelibrary.com]

The phages isolated in this study ranged in size from 50.34 kb (vB_Eco_mar002J1) to 167.77 kb (vB_Eco_mar005P1), with between 78 (vB_Eco_mar001J1) and 267 (vB_Eco_mar005P1) predicted genes per genome. While vB_Eco_mar004NP2 and vB_Eco_mar003J3 are both part of the genus *T5virus*, their genome sizes were 107.6 and 115.47 kb, respectively (Supporting Information Table S4). This ~7.8 kb difference in genome size is a reflection of the diversity of phages within the genus *T5virus*, whereby the core gene content constitutes a small proportion of the total gene content. For vB_Eco_mar004NP2 and vB_Eco_mar003J3, the core‐gene content is 10.7% and 10.2% of the total genes, respectively. Genomic comparisons across the genus *T5virus* reveal multiple regions that are present in some phages and not others (Fig. 4, Supporting Information Fig. S4, and Supporting Information Table S2). In contrast, the core gene content of *Rb69virus* constitutes a much larger proportion. In vB_Eco_mar005P1, this is 63% of the total genes, with greater conservation in gene content across the genus (Supporting Information Fig. S5 and Supporting Information Table S1). The phages vB_Eco_mar001J1, vB_Eco_mar002J2 and vB_Eco_swan01 only had four core genes with other members of the *Tunavirinae,* all of which are hypothetical proteins (Supporting Information Table S3). Comparison of phages just within the proposed new genus *psFunavirus* reveals a conservation in gene content and phylogeny (Supporting Information Fig. S6).

### *TEM*

**Figure 5:**
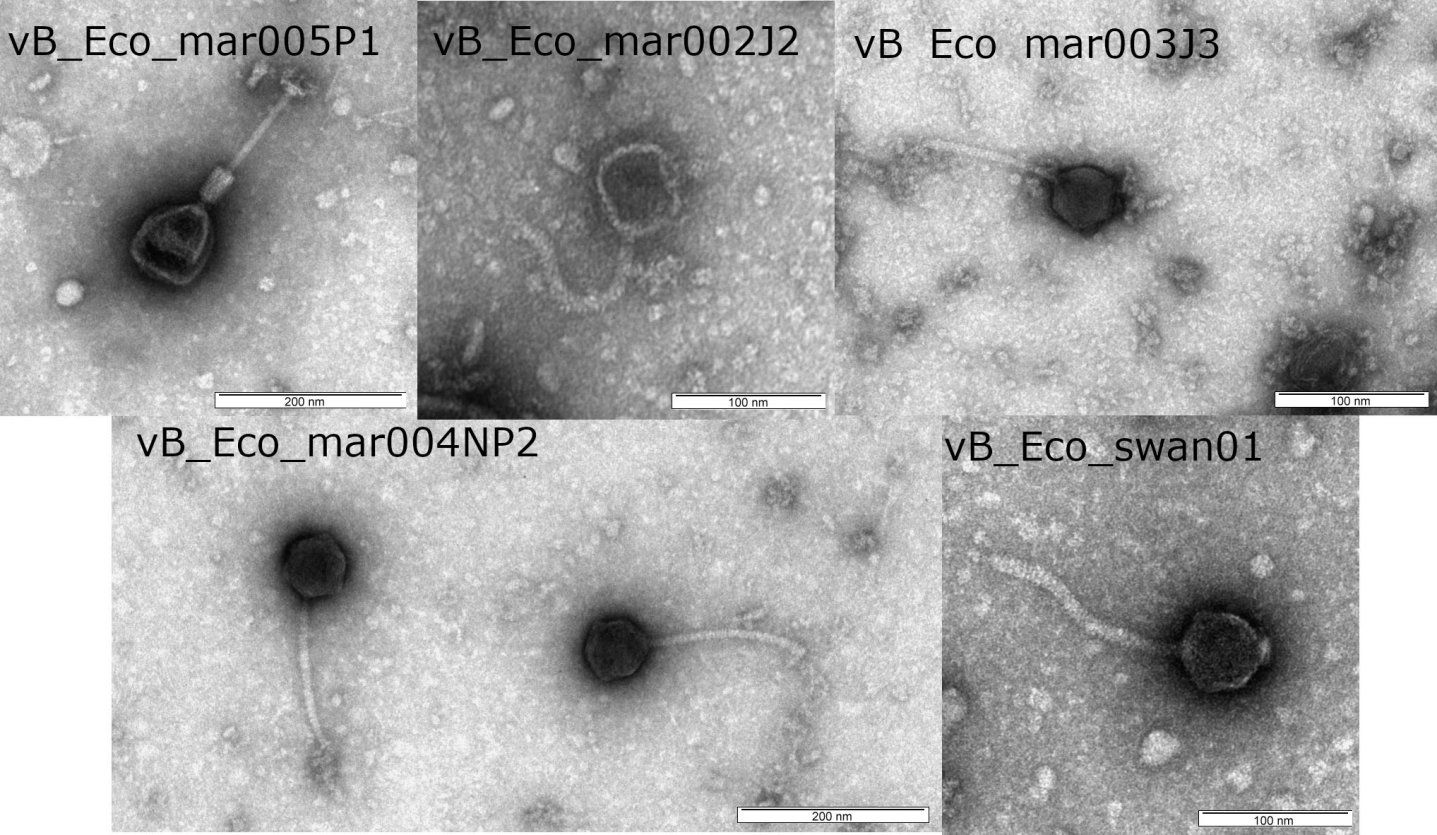
Morphology of phage isolates**.** Phages vB_Eco_swan01, vB_Eco_mar005P1, vB_Eco_mar002J2, vB_Eco_mar003J3, vB_Eco_mar004NP2 were stained with 2% (w/v) uranyl acetate and imaged in a JEOL JEM‐1400 TEM with an accelerating voltage of 100 kV.

TEM analysis confirmed vB_Eco_swan01, vB_Eco_mar005P1, vB_Eco_mar002J2, vB_Eco_mar003J3 and vB_Eco_mar004NP2 were all members of the order *Caudovirales* (Fig. 5, Table 2), which contains all known tailed bacteriophages. Furthermore, phages vB_Eco_mar002J2, vB_Eco_mar003J3, vB_Eco_mar004NP2 and vB_Eco_swan01 were observed to have long non‐contractile tails with a polyhedral head, which are signatures of the family *Siphoviridae*, thus confirming the phylogenetic analysis. The head length: width ratio further classified the phages within subgroup B1 (Ackermann and Krisch, 1997). Phage vB_Eco_mar005P1 was also observed to have a long contractile tail, with tail fibres clearly observable and a distinct prolate head which allows classification within sub group A2 of the *Myoviridae* (Ackermann and Krisch, 1997) (Fig. 5, Table 2).

**Table 2:** Morphological and lytic properties of representative phages.

Phage isolate	Burst size	Latent period	Eclipse period	Head width (nm)	Head length (nm)	Tail length (nm)	Tail width (nm)	Sub group	Taxonomy
vB_Eco_swan01	78 ± 9	15	9	53 ± 2	56 + −1	154 ± 10	10 ± 1	B1	*Siphoviridae, Tunavirinae*
vB_Eco_mar002J2	51 ± 17	12	9	55 ± 4	56 + −4	143 ± 13	11 ± 1	B1	*Siphoviridae, Tunavirinae*
vB_Eco_mar004NP2	193 ± 26	33	20	66 ± 2	71 + −5	176 ± 9	10 ± 1	B1	*Siphoviridae, T5virus*
vB_Eco_mar003J3	76 ± 22	40	26	67 ± 5	70 + −5	185 ± 19	9 ± 1	B1	*Siphoviridae, T5virus*
vB_Eco_mar005P1	31 ± 9	14	23	86 ± 6	111 + −11	121 ± 7	20 ± 3	A2	*Myoviridae, Tevenvirinae, Rb69virus*

### *Proteomic characterization*

As with most phages, the majority of the genes predicted within each genome encode hypothetical proteins with unknown function. In order to identify further structural proteins or proteins that may be contained within the capsid, proteomic analysis of representative phages was carried out using electrospray ionization mass spectrometry (ESI‐MS/MS). The number of identified proteins per phage was five, five, seven and eight for phages vB_Eco_mar005P1, vB_Eco_swan01, vB_Eco_mar003J3, and vB_Eco_mar004NP2, respectively (Supporting Information Table S5a). This allowed the confirmation of two annotated structural proteins (SWAN_00017 and SWAN_00019) and the identification of a further three structural proteins (SWAN_00025, SWAN_00026 and SWAN_00027). Based on the core‐gene analysis this allowed annotation of orthologues of SWAN_00017, SWAN_00019 and SWAN_00025 in vB_Eco_mar001J1, vB_Eco_mar002J2 and SECphi27, and SWAN_00026 and SWAN_00027 in vB_Eco_mar001J1 and vB_Eco_mar002J2.

For phage vB_Eco_mar005P1, five proteins were identified three of which confirmed annotations as structural proteins (MAR005P1_00047, MAR005P1_00051 and MAR005P1_00054) all of which are core genes to phages within the genus *Rb69virus*, along with an ADP‐ribosyltransferase protein (MAR005P1_00076) that is packaged within the phage capsid. An additional structural protein (MAR005P1_00015) was confirmed that was previously annotated as a hypothetical protein, which is also found in phages vB_Eco_mar005P1, vB_Eco_mar006P2, vB_Eco_mar007P3, vB_Eco_mar008P4 and vB_Eco_mar009P5.

Both phages vB_Eco_mar004NP2 and vB_Eco_mar003J3 are part of the genus *T5virus*, although distantly related. For phage vB_Eco_mar004NP2, eight proteins were detected that confirmed their annotation as various structural components of the capsid and tail (Supporting Information Table S5a). For proteins MAR003J3_00086 and MAR003J3_00094–97, the orthologous proteins in vB_Eco_mar004NP2 were also detected. Proteins MAR004NP2NP2_00151, MAR004NP2_00157 and MAR004NP2_00160 were only detected in vB_Eco_mar004NP2. However, orthologous proteins were detected in vB_Eco_mar003J3 through core‐gene analysis. Protein MAR003J3_00081, which is a putative tail fibre, was only detected in vB_Eco_mar003J3, with no orthologue in vB_Eco_mar004NP2 based on core‐gene analysis (Supporting Information Table S2 and Supporting Information Fig. S4).

### *Phage infection parameters*

The burst size, latent period and eclipse period for representative phage isolates was also determined (Table 2). There was considerable variation in these parameters across all isolates, with burst size ranging from 31 (vB_Eco_mar005P1) to 192 (vB_Eco_mar004NP2) (Table 2). Similar variation was observed for the latent period varying from 12 min (vB_Eco_mar002J2) to 40 min (vB_Eco_mar003J3), while the eclipse period ranged from 9 min (vB_Eco_swan01 & vB_Eco_mar002J2) to 26 min (vB_Eco_mar003J3). For phages vB_Eco_mar003J3 and vB_Eco_mar004NP2 that are part of the same genus (T*5virus)*, there was considerable variation in all three parameters, with the burst size of vB_Eco_mar004NP2 (193) double that of vB_Eco_mar003J3 (76).

### *Phage host range*

The host range of representative phage isolates was determined using a range of bacterial hosts via a spot test assay (Supporting Information Table S6). Phylogenetic analysis highlighted that the isolated coliphages were often closely related to phages that are known to infect other Enterobacteriaceae, including *Klebsiella* and *Salmonella* (Figs 1, 2, and 3). For this reason, the host range of these phage was also tested against other Enterobacteriaceae. Phage vB_Eco_mar005P1, a representative of the genus *Rb69virus*, was only able to infect its host of isolation (*E. coli* MG1655), whereas phages of the genus *T5virus* and subfamily *Tunavirinae* were capable of infecting between five and eight strains (Supporting Information Table S6). While vB_Eco_mar002J2 was found to infect the greatest number of strains (8), this was limited to strains of *E. coli*, *Klebsiella pneumoniae*, *and Klebsiella oxytoca*, whereas vB_Eco_mar004NP2 could also infect *Salmonella typhimurium,* but fewer strains of *E. coli*.

### *Detection in viral metagenomes*

The presence of these new coliphage species in viral metagenomes was investigated using existing metagenomics databases. The Baltic virome data set was chosen as it contains both DNA sequence data and RNA expression data (Zeigler‐Allen *et al.,* 2017). Based on the criteria of 75% genome coverage and 90% identity (Roux *et al.,* 2017), coliphage were not detectable in this viral metagenomics data set. We then searched for evidence of gene expression from these phages using the much larger Baltic virome metatranscriptomics data set, using cyanophage Syn9 as a control, since it has previously been reported in this data set (Zeigler‐Allen *et al.,* 2017). The majority of samples showed the expression of cyanophage Syn9 genes, as previously reported (Zeigler‐Allen *et al.,* 2017). Interestingly, the expression of genes from coliphage NP2 and RB69 (Supporting Information Fig. S7) was also detected, in samples GS852 and GS677, respectively. These samples, GS852 and GS677, were collected from low‐salinity surface waters (Zeigler‐Allen *et al.,* 2017). The reads mapping to these coliphages were further analysed by BLASTn. As well as possessing similarity to the coliphage they mapped against, these reads were also similar to other closely related coliphages and an unannotated prophage region in *E. coli* genomes, confirming they are transcripts from coliphages or very closely related enterobacterial phages.

## Discussion

Using *E.coli* MG1655, we were able to isolate and characterize ten phages (six unique phages) from coastal marine waters and one from a freshwater pond. The titre of coliphages in all water samples was extremely low (range 0.0125 pfu ml^−1^‐0.28 pfu ml^−1^). This low abundance is lower than previous reports of coliphages in coastal environments that are around 1 × 10^2^ pfu ml (Dutka *et al.,* 1987; Janelidze *et al.,* 2011; Burbano‐Rosero *et al*., 2011). This may be linked to water quality, since coliphage abundance is known to be linked to faecal contamination. Alternatively, the time of sampling may be a factor, since previous work has found there are distinct seasonal patterns in coliphage abundance (Janelidze *et al.,* 2011), or our choice of *E. coli* host strain, which has also been shown to affect abundance estimates (Havelaar and Hogeboom, 1983; Jofre, 2009; Muniesa *et al.,* 2013). Despite this low abundance, it was still possible to isolate coliphages to further characterize their genetic diversity, which was the focus of this study.

Given the small number of phages isolated and sequenced, there was a surprising amount of phylogenetic diversity (Figs 1, 2, 3). Five species of coliphage were identified in the 10 phages isolated. Phages vB_Eco_mar005P1, vB_Eco_mar006P2 and vB_Eco_mar008P4 were identical, with vB_Eco_mar009P5 and vB_Eco_mar007P3 only differing by a few SNPs. This similarity is probably due to the enrichment method, which has enriched for a single phage that has then proliferated in the enrichment and been reisolated. It is also possible that seawater provides a selection pressure and only certain types of coliphages are able to survive. Phages vB_Eco_mar001J1 and vB_Eco_mar002J2 also had identical genome sequences despite being independently isolated and represent a novel species. The remaining phages vB_Eco_mar003J3, vB_Eco_mar004NP2 and vB_Eco_swan01 were all unique and also represent new species.

Phages infecting *Escherichia* account for ~7% of all phages sequenced to date. To discover a novel genus from the sequencing of a just small number of coliphages further highlights the vast diversity of phages present in the environment and how much more there is to be discovered. To accurately place phages in the context of current phage taxonomy, we identified core genes and used the GET_PHYLOMARKERS pipeline to select the most appropriate gene for phylogenetic reconstruction, that is, a gene that does not show signs of recombination, a process that could lead to inaccurate branch lengths (Didelot and Maiden, 2010). Our phylogenetic analysis of phage genomes using selected marker genes was congruent with current classifications of phage species. Some of these classifications are originally based on historical phenotypic data such as the inability of phage RB69 to recombine with phage T4 leading to its classification as a separate species (Russell, 1967). Recently, this inability to recombine with phage T4 DNA was postulated to be caused by the arabinosyl modification of DNA in RB69, likely caused by a novel glucosyltransferase present in RB69 but not T4 (Thomas *et al.,* 2018). In this study, the gene thought to encode a putative arabinosyltransferase (Thomas *et al.,* 2018) was found to be core in all members of the genus *Rb69virus*. Whether the phage isolated in this study also glycosylate their DNA in a similar manner to RB69 remains to be determined. However, the genes thought to be responsible for it are clearly a signature of this genus.

While the phylogenetic analysis was congruent with currently defined species within the *T5virus* and *Rb69virus* genera, combining this phylogenetic analysis with ANI data demonstrated that using an ANI value >95% was insufficient to delineate species that were consistent with the observed phylogeny when additional phage from this study, and those present in GenBank but having undefined species, were added. Phages that formed clearly distinct clades had an ANI >95% with phages outside of the phylogenetic clades, suggesting 95% ANI is insufficient to discriminate between species for some genera. We therefore suggest an ANI of 97% should be used to discriminate phage within the genera *T5virus* and *Rb69virus*, which has previously been used for the demarcation of phage species within the genus *Seuratvirus* (Sazinas *et al.,* 2017).

In the context of coliphages in general, the phage isolated in this study and subsequent analysis has further expanded our knowledge of the genetic diversity of coliphages and identified new taxonomic groups. The closest relatives of phages isolated in this study were all other coliphages or phages infecting other Enterobacteriaceae. Exactly what constitutes a ‘coliphage’ is unclear, since, as seen from this study, coliphages can also infect other *Enterobacteriaceae*. Comparative genome analysis revealed a large difference in the genomic content of phages, with phages of the genus *Rb69virus* having a large core‐genome while those of the genus *T5virus* have a much smaller core‐genome (Fig 4 and Supporting Information Fig. S5). Whether this is due to different phage species having more flexible genomes that allows frequent recombination, or a reflection of the diversity of hosts used to isolate T5‐like phages, requires further investigation.

Proteomic analysis of the representative phages resulted in a relatively small number of proteins being detected per phage. Despite this, it was still possible to confirm the annotation of structural proteins and identify new structural proteins in phage vB_Eco_mar005P1 and vB_Eco_swan01. Combined with the core‐gene analysis, it confirmed the annotation of a large number of genes across all phage isolates as structural proteins. In addition, the detection of an ADP‐ribosyltransferase in vB_Eco_mar005P1 suggests that the carriage of this protein is common to phages in the genus *Rb69virus* and presumably acts similarly to the ADP‐ribosyltransferase carried by phage T4, in modifying the host RNA polymerase for early gene transcription (Koch *et al.,* 1995; Miller *et al.,* 2003). For phage vB_Eco_mar003J3, a putative tail fibre gene (MAR003J3_00081) was detected for which there is no orthologue in vB_Eco_mar004NP2.

The gene encoding MAR003J3_00081 is an orthologue of *ltfA* in phage DT57C and DT571/2 which with l*tfB* encode for L‐shaped tail fibres that allow attachment to different O‐antigen types. This arrangement of two genes encoding the L‐shaped tail fibres is different from T5, which encodes the L‐shaped tail fibres in a single gene (Golomidova *et al.,* 2016; Nobrega *et al.,* 2018). vB_Eco_mar003J3 contains orthologues of both *ltfA* and *ltfB*, suggesting that it too uses two gene products for L‐shaped tail fibres, whereas vB_Eco_mar004NP2 only contains an orthologue of *ltfB* (MAR004NP2_00162) and does not contain an orthologue of the single gene used by T5 (*ltf*). Comparison of the genomic context of the region of *ltfB* in vB_Eco_mar004NP2 reveals two genes immediately upstream of *ltfB* that do not have orthologues in vB_Eco_mar003J3, one of which likely encodes a protein to form the L‐shaped tail fibre with the product of *lftB*. Similarly, there are two genes upstream of *ltfAB* in vB_Eco_mar003J3 that are absent in vB_Eco_mar004NP2. However, immediately beyond this the genome contains 10 genes either side of these genes that are present in the same order in both genomes (Supporting Information Fig. S4 and Supporting Information Table S2). Given the observed difference in host range between phages vB_Eco_mar003J3 and vB_Eco_mar004NP2, we speculate that it is the differences in the region that contains tail fibre genes that is likely responsible and contributes to the ability of vB_Eco_mar004NP2 to infect multiple genera of Enterobacteriaceae.

Differences in the properties of vB_Eco_mar003J3 and vB_Eco_mar004NP2 were also observed in terms of their replication parameters, with vB_Eco_mar004NP2 having a burst size (193) twice that of vB_Eco_mar003J3 (76). It has previously been reported that phage chee24, which is also part of the genus *T5virus*, has a burst size of 1000 and a latent period of 44 min (Sváb *et al.,* 2018). However, this number does appear to be an outlier because other *T5virus* phages such as phage T5 and chee30 have burst sizes of ~77 and ~44, respectively, suggesting considerable variation within the genus.

In comparison, there was similar variation in the burst size of phages within the genus *Rb69virus*, with vB_Eco_mar005P1 having a burst size that is very similar to the reported burst size of 31 for phage RB69, but smaller than the burst size of 96 for phage APCE01 (Dalmasso *et al.,* 2016). Whether the lytic properties of phages does correlate with phylogeny requires more data than is currently available and would require standardized growth conditions for like‐for‐like comparisons, given it is known differences in temperature can influence burst size.

Detection of reads mapping to coliphages in the Baltic virome metatranscriptomics data set was surprising given coliphage are not thought to actively replicate in seawater (Jofre, 2009), and that they were not detected in the metagenomics data set. However, the latter observation may be explained by the substantially larger amount of metatranscriptomics data from Illumina sequencing (138 Gb) in this Baltic virome data set compared to the 454 metagenomic sequencing data (~7.9 Gb).

## Conclusions

We have begun to elucidate for the first time the genomic diversity of coliphage within seawater, identifying phages that represent several novel taxa, further expanding the diversity of phages that are known to infect *E. coli*. Furthermore, the analysis and identification of core‐genes and selection of genes suitable for phylogenetic analysis provides a framework for the future classification of phages in the genera *Rb69virus*, *T5virus*, and subfamily *Tunavirinae*. We further suggest that an ANI of >95% is not suitable for the delineation of species within the genera *Rb69virus* and *T5virus* and that a value of >97% ANI should be used. Characterization of phage replication parameters and host range further reinforces that morphologically similar phage can have diverse replication strategies and host ranges. While we are cautious about the detection of coliphage transcripts in seawater metatranscriptomes, the most parsimonious explanation is that coliphage are actively replicating, an observation that certainly warrants further investigation.

## Materials and methods

### *Phage isolation*

*Escherichia coli* MG1655 was used as the host for both phage isolation and phage characterization work, as it has previously been used to isolate a wide diversity of coliphages (Smith *et al.,* 2015; Sazinas *et al.,* 2016, 2017; Michniewski *et al.,* 2017). *E .coli* MG1655 was cultured in LB broth at 37°C with shaking (200 rpm). Seawater samples were collected from United Kingdom and Polish coastal waters (see Table 1), filtered through a 0.22 μm pore‐size polycarbonate filter (Sarstedt) and stored at 4°C prior to use in plaque assays. Plaque assays were undertaken within 24 h of collecting these samples. Phages were initially isolated and enumerated using a simple single layer plaque assay (Van Twest and Kropinski, 2009). However, where this was unsuccessful, a modified plaque assay was used that allowed a greater volume of water to be added. Briefly, filtered seawater was mixed with CaCl_2_ to a final concentration of 1 mM followed by addition of *E. coli* MG1655 cells at a 1:20 ratio and incubating the mixture at room temperature for 5 min. Subsequently, samples were mixed with molten LB agar at a 1:1 ratio, final concentration 0.5% (w/v). Agar plates were incubated overnight at 37°C and checked for the presence of plaques. For samples in which no coliphage were detected, an enrichment procedure was carried out. Briefly, 20 ml of filtered seawater was mixed with 20 ml LB broth and 1 ml *E. coli* MG1655 (OD600 = ~0.3 i.e. mid‐exponential phase) and incubated overnight at 37°C, followed by filtration through a 0.22 μm pore‐size filter. Phages from this enriched sample were then isolated using the standard plaque assay procedure. Three rounds of plaque purification were used to obtain clonal phage isolates (Van Twest and Kropinski, 2009) .

### *Host range*

Host range for each phage was determined by spot assay. Briefly, 1 ml of mid‐log phase bacteria was mixed with 5 ml of molten 0.5% (w/v) LB agar, poured onto a base layer of 1% (w/v) LB agar layer and incubated at 37°C for 1 h. Subsequently, phage stocks (~1 × 10^9^ pfu/ml) underwent serial dilution (down to 10^−8^), and 10 μl of each dilution was spotted on a bacterial lawn. The host was deemed susceptible to phage infection when the size of the clearing consistently decreased with the dilution, to the point that single plaques could be observed.

### *Genome sequencing*

Phage DNA was prepared using a previously established method (Rihtman *et al.,* 2016). DNA was quantified using Qubit and 1 ng DNA used as input for NexteraXT library preparation following the manufacturer's instructions. Sequencing was carried out using a MiSeq platform with V2 (2 × 250 bp) chemistry. Fastq files were trimmed with Sickle v1, using default parameters (Joshi *et al.,* 2011). Genome assembly used SPAdes v3.7 with the careful option (Bankevich *et al.,* 2012). Reads were then mapped back against the resulting contig with BWA MEM v0.7.12 (Li, 2013) and SAM and BAM files manipulated with SAMtools v1.6 to determine the average coverage of each contig (Li, 2013). If the coverage exceeded 100× then the reads were subsampled and the assembly process repeated, as high coverage is known to impede assembly (Rihtman *et al.,* 2016). Phage genomes were then annotated with Prokka using a custom database of all phage genomes that had previously been extracted from Genbank (Seemann, 2014). Further annotation was carried out using the pVOG database to annotate any proteins that fall within current pVOGS using hmmscan (Eddy, 2011; Grazziotin *et al.,* 2017). Raw sequence data and assembled genomes were deposited in the ENA under project accession number PRJEB28824.

### *Bioinformatics and comparative genomics*

A MASH database was constructed of all complete bacteriophage genomes available at the time of analysis (~ 8500, April 2018) using the following mash v2 settings “–s 1000” (Ondov *et al.,* 2016) (see Supporting Information). This database was then used to identify related genomes based on MASH distance, which has previously been shown to be equivalent to ANI (Ondov *et al.,* 2016). From this initial set of genomes, single marker genes were used for initial placement of the newly isolated phages on a phylogenetic tree, using IQ‐TREE. Following this, a more detailed analysis of the most closely related genomes was carried out. Phage genomes that were found to be similar were reannotated with Prokka to ensure consistent gene calling between genomes for comparative analysis (Seemann, 2014). Core genome analysis was carried out with ROARY using “‐‐e ‐‐mafft ‐p 32 –i 90” as a starting point for analysis (Page *et al.,* 2015). These parameters were adjusted as detailed in the text. The optimal phylogenetic markers were determined using the GET_PHYLOMARKERS pipeline, with the following settings “‐R1 –t DNA” (Vinuesa *et al.,* 2018). Average nucleotide identity was calculated using autoANI.pl (Davis II *et al.,* 2016). Phylogenetic analysis was carried out using IQ‐TREE (Nguyen *et al.,* 2015), with models of evolution selected using model test (Posada and Crandall, 1998); trees were visualized in ITOL (Letunic and Bork, 2007).

### *One‐step growth experiments*

Phage growth parameters (burst size, eclipse and latent period) were determined by performing one‐step growth experiments as described by Hyman and Abedon (2009), with free phages being removed from the culture by pelleting the host cells via centrifugation at 10,000 g for 1 min, removing the supernatant and resuspending cells in fresh medium (Hyman and Abedon, 2009). Three independent replicates were carried out for each experiment.

### *TEM*

Representative phages, as determined from genome sequencing, were imaged using a Transmission electron microscope (TEM) as follows: 10 μl of high titre phage stock was added to a glow discharged formvar copper grid (200 mesh), left for 2 min, wicked off, and 10 μl of water added to wash the grid prior to being wicked off with filter paper. Ten microliter of 2% (w/v) uranyl acetate stain was added to the grid and left for 30 s, prior to its removal. The grid was air dried before imaging using a JEOL JEM‐1400 TEM with an accelerating voltage of 100 kV. Digital images were collected with a Megaview III digital camera using iTEM software. Phage images were processed in ImageJ using the measure tool and the scale bar present on each image to obtain phage particle size (Rasband, 2016). Measurements are the average of at least 13 phage particles.

### *Preparation of viral proteomes for nanoLC‐MS/MS and data analysis*

Prior to proteomics high‐titre phage stocks were purified using CsCl density gradient centrifugations at 35,000 g for 2 h at 4 °C. Subsequently, 30 μl of concentrated phage was added to 10 μl NuPAGE LDS 4X sample buffer (Invitrogen) heated for 5 min at 95°C and analysed by SDS‐PAGE as described (Kaur *et al.,* 2018). Polyacrylamide gel bands containing all phage proteins were excised and standard in‐gel reduction with iodoacetamide and trypsin (Roche) proteolysis was performed prior to tryptic peptide extraction (Kaur *et al.,* 2018). Samples were separated and analysed by means of a nanoLC‐ESI‐MS/MS using an Ultimate 3000 LC system (Dionex‐LC Packings) coupled to an Orbitrap Fusion mass spectrometer (Thermo Scientific, Waltham, MA, USA) with a 60 min LC separation on a 25 cm column and settings as described previously (Kaur *et al.,* 2018). Compiled MS/MS spectra were processed using the MaxQuant software package (version 1.5.5.1) for shotgun proteomics (Cox and Mann, 2008). Default parameters were used to identify proteins (unless specified below), searching an in‐house‐generated database derived from the translation of phage genomes. Firstly, a six reading frame translation of the genome with a minimum coding domain sequence (CDS) cut‐off of 30 amino acids (i.e. stop‐to‐stop) was used to search for tryptic peptides. Second, the search space was reduced by using a database containing only CDS detected in the first database search, again, looking for tryptic peptides. Finally, the reduced CDS database was also searched using the N‐terminus semi‐tryptic digest setting to find the protein N‐terminus. Analysis was completed using Perseus software version 1.6.0.7 (Tyanova *et al.,* 2016). All detected peptides from all three analyses are compiled in Supporting Information Table S5b. Only proteins detected with two or more non‐redundant peptides were considered.

## Supporting information


**Appendix S1**: Supporting Information

**Table S1.** Core‐genes, ANI and genes used for phylogenetic analysis of phages within the genus *RB69virus*. All phages were re‐annotated to ensure consistent gene calling. ANI was calculated using autoANI. See attached excel file.

**Table S2.** Core‐genes, ANI, and genes used for phylogenetic analysis of phages within the genus *T5*virus. All phages were re‐annotated to ensure consistent gene calling. ANI was calculated using autoANI. See attached excel file.

**Table S3.** Core‐genes, ANI, and genes used for phylogenetic analysis of phages within the subfamily *Tunavirinae*. ANI was calculated using autoANI. See attached excel file.

**Table S4.** Genome properties of bacteriophages: vB_Eco_mar004NP2, SWAN, vB_Eco_mar002J1, vB_Eco_mar002J2, vB_Eco_mar003J3, vB_Eco_mar005P1, vB_Eco_mar005P2, vB_Eco_mar005P3vB_Eco_mar005P4, vB_Eco_mar005P5 and vB_Eco_mar005P6. See attached excel file.

**Table S5a.** Proteomic analysis of phages vB_Eco_swan01, vB_Eco_mar005P1, vB_Eco_mar002J2, vB_Eco_mar003J3 and vB_Eco_mar004NP2. See attached Word document.
**Table S5b.** Peptides detected in phages vB_Eco_swan01, vB_Eco_mar005P1, vB_Eco_mar002J2, vB_Eco_mar003J3 and vB_Eco_mar004NP2. See attached excel file.
**Table S6.** Host range of coliphages vB_Eco_swan01, vB_Eco_mar005P1, vB_Eco_mar002J2, vB_Eco_mar003J3 and vB_Eco_mar004NP2 against Enterobacteriaceae hosts. Infected hosts are marked with a black box and those that are not infected with a ‐. see attached Word document.

**Figure S1.** Phylogenetic analysis of phages within the genus *RB69virus*. The tree is based on the nucleotide sequence of the major capsid protein (*g23*), using a TIM2 + F + R5 model of evolution, with 1000 bootstrap replicates using IQTREE (Nguyen *et al*., 2015). The phages included in the tree are vB_MmoM_MP1 (acc:KX078569), PS2 (acc:KJ025957), phiR1‐RT (acc:HE956709), vB_YenM_TG1 (acc:KP202158), JSE (acc:EU863408), *Aeromonas* phage 25 (acc:DQ529280), 44RR2 (acc:AY375531), 44RR2.8 t.2 (acc:KY290948), *Aeromonas* phage 31.2 (acc:KY290951), *Aeromonas* phage 31 (acc:AY962392), Riv‐10 (acc:KY290957), L9‐6 (acc:KY290956), SW69‐9 (acc:KY290958), Acj9 (acc:HM004124), Ac42 (acc:HM032710), Acj61 (acc:GU911519), Merlin (acc:KT001915), Moon (acc:KM236240), CF1 (acc:MG250484), STML‐198 (acc:JX181825), Melville (acc:MF957259), vB_SnwM_CGG4‐1 (acc:KU867307), KP1 (acc:MG751100), PKO111 (acc:KR269720), JD18 (acc:KT239446), vB_Kpn_F48 (acc:MG746602), PG7 (acc:KJ101592), Pet‐CM3‐4, (acc:LT614807), CC31 (acc:GU323318), JS10 (acc:EU863409), vB_EcoM_VR5 (acc:KP007359), SP18 (acc:GQ981382), vB_EcoM_VR20 (acc:KP007360), vB_EcoM_VR7 (acc:HM563683), vB_EcoM_VR25 (acc:KP007361), vB_EcoM_VR26 (acc:KP007362), PEi20 (acc:AP014714), PEi26 (acc:AP014715), CHI14 (acc:MF036690), CBH8 (acc:MF036691), X20 (acc:MF036692), PM2 (acc:KF835987), JS98 (acc:EF469154), IME08 (acc:HM071924), MX01 (acc:KU878969), WG01 (acc:KU878968), QL01 (acc:KT176190), Bp7 (acc:HQ829472), E. coli O157 typing phage 3 (acc:KP869101), E. coli O157 typing phage 6 (acc:KP869104), RB69 (acc:AY303349), SHSML‐52‐1 (acc:KX130865), vB_EcoM_PhAPEC2 (acc:KF562341), phiC120 (acc:KY703222), APCEc01 (acc:KR422352), vB_Eco_mar005P1 (acc:LR027390), Shf125875 (acc:KM407600), ST0 (acc:MF044457), HX01 (acc:JX536493), vB_EcoM_JS09 (acc:KF582788), HP3 (acc:KY608965), RB59 (acc:KM607003), RB55 (acc:KM607002), T4 strain wild (acc:KJ477684), T4 (acc:AF158101), slur07 (acc:LN881732), PE37 (acc:KU925172), vB_EcoM_UFV13 (acc:KU867876), T4T (acc:HM137666), T4 strain GT7 (acc:KJ477686), T4 strain 147 (acc:KJ477685), ime09 (acc:JN202312), vB_CroM_CrRp10 (acc:MG775043), Shfl2 (acc:HM035025), RB14 (acc:FJ839692), vB_EcoM_112 (acc:KJ668714), RB51 (acc:FJ839693), RB68 (acc:KM607004), vB_EcoM_ACG‐C40 (acc:JN986846), SHFML‐26 (acc:KX130862), EC121 (acc:MF001359), RB32 (acc:DQ904452), RB33 (acc:KM607001), pSs‐1 (acc:KM501444), SH7 (acc:KX828711), PST (acc:KF208315), SG1 (acc:MF001354), Sf22 (acc:MF158045), EC04 (acc:MF001360), slur03 (acc:LN881728), slur14 (acc:LN881736), slur08 (acc:LN881733), Sf21 (acc:MF327007), SHBML‐50‐1 (acc:KX130864), KPN1 (acc:KX452694), KPN5 (acc:KX452698), SF25 (acc:MF327009), Sf24 (acc:MF327008), ECML‐134 (acc:JX128259), HY01 (acc:KF925357), PEC04 (acc:KR233165), UFV‐AREG1 (acc:KX009778), RB3 (acc:KM606994), RB6 (acc:KM606996), RB9 (acc:KM606998), RB10 (acc:KM606999), RB7 (acc:KM606997), RB5 (acc:KM606995), RB27 (acc:KM607000), wV7 (acc:HM997020), E. coli O157 typing phage 7 (acc:KP869105), AR1 (acc:AP011113), Sf23 (acc:MF158046), SHFML‐11 (acc:KX130861), HY03 (acc:KR269718), vB_EcoM‐fFiEco06 (acc:MG781190), vB_EcoM‐fFiEco01 (acc:MG781191), YUEEL01 (acc:KY290975), CF2 (acc:KY608967), phiD1 (acc:HE956711), slur02 (acc:LN881726), slur13 (acc:LN881737), slur11 (acc:LN881734), slur04 (acc:LN881729). Phages in the genus ***RB69virus*** are coloured in blue

**Figure S2.** Phylogenetic analysis of phages within the genus *T5virus*. The phylogenetic tree is based on the nucleotide sequence of the gene encoding DNA polymerase, using a TIM2 + F + R3 model of evolution, with 1000 bootstrap replicates using IQTREE (Nguyen *et al.,* 2015). The phages included in the tree are HTVC010P (acc:NC_020481), phiR201 (acc:HE956708), saus132 (acc:MF431737), poul149 (acc:MF431738), saus176N (acc:MF431741), chee158 (acc:MF431739), chee130_1 (acc:MF431736), cott162 (acc:MF431740), vB_Eco_mar003J3 (acc:LR027389), Stitch (acc:KM236244), EPS7 (acc:CP000917), BSP22A (acc:KY787212), SH9 (acc:MF001363), 100268_sal2 (acc:KU927497), 118970_sal2 (acc:KX017521), LVR16A (acc:MF681663), APCEc03 (acc:KR422353), slur09 (acc:LN887948), SP3 (acc:MG387042), bV_EcoS_AKFV33 (acc:HQ665011), SPC35 (acc:HQ406778), SP01 (acc:KY114934), SSP1 (acc:KY963424), vB_Eco_mar004NP2 (acc:LR027384), phiLLS (acc:KY677846), Shivani (acc:KP143763), SHSML‐45 (acc:KX130863), OSYSP (acc:MF402939), T5, st0 del mutant (acc:AY692264), T5 (acc:AY543070), T5,ATCC 11303‐B5 (acc:AY587007), DT57C (acc:KM979354), DT571/2 (acc:KM979355), vB_EcoS_FFH1 (acc:KJ190157), pork27 (acc:MF431731), poul124 (acc:MF431735), saus47N (acc:MF431733), saus111K (acc:MF431734), chee24 (acc:MF431730), pork29 (acc:MF431732).

**Figure S3.** Phylogenetic analysis of phages within the subfamily *Tunavirinae*. The tree is based on the nucleotide sequence of the terminase gene, using a TIM2 + F + R3 model of evolution, with 1000 bootstrap replicates using IQTREE (Nguyen *et al.,* 2015). The phages included in the tree are phiEt88 (acc:FQ482085), JMPW2 (acc:KU194205), T1 (acc:NC_005833), JMPW1 (acc:KU194206), Shfl1 (acc:NC_015456), ADB‐2 (acc:NC_019725), pSf‐2 (acc:NC_026010), Esp2949‐1 (acc:NC_019509), vB_Eco_mar001J1 (acc:LR027388), vB_Eco_mar002J2 (acc:LR027385), pSf‐1 (acc:KC710998), vB_Eco_swan01 (acc:LT841304), SECphi27 (acc:LT961732), SP126 (acc:KC139513), TLS (acc:NC_009540), YSP2 (acc:MG241338), Stevie (acc:NC_027350), PKP126 (acc:NC_031053), F20 (acc:JN672684), KLPN1 (acc:KR262148), 1513 (acc:KP658157), KP36 (acc:NC_029099), MezzoGao (acc:MF612072), Sushi (acc:KT001920), GML‐KpCol1 (acc:MG552615), Rtp (acc:NC_007603), vB_Eco_ACG‐M12 (acc:NC_019404), vB_EcoS_Rogue1 (acc:NC_019718), phiJLA23 (acc:KC333879), C119 (acc:KT825490), e4/1c (acc:NC_024210), vB_EcoS_AKS96 (acc:NC_024789), vB_EcoS_AHP42 (acc:NC_024793), bV_EcoS_AHP24 (acc:KF771236), vB_EcoS_AHS24 (acc:NC_024784).

**Figure S4.** Genomic alignment of phages vB_Eco_mar004NP2 and vB_Eco_mar003J3. Genomes were re‐ordered from the gene encoding the terminase large subunit to allow ease of comparison. Genomes were compared with EasyFig (Sullivan *et al*., 2011) using blastn (minimum length 100 e‐value 0.001). Genes detected by mass spectrometry are shaded in yellow.

**Figure S5.** Comparative genomic analysis of the genus *Rb69virus*. All phages were compared to the type phage RB69 (accession: AY303349) using BRIG (Alikhan *et al.,* 2011). From the inside out, each ring represents a blastn similarity (e‐value 0.001) to phage RB69. The darker the shading within each ring, the higher the similarity. The outer two rings mark the genes and annotation as extracted from the Genbank file (AY303349).

**Figure S6.** Comparative analysis of the proposed genus *psFunavirus*. All genomes were re‐ordered with the gene encoding the terminase subunit as a starting point. Genomes were compared with blastn (minimum length − 100, e‐value −0.001) using EasyFig. Genomes were compared in a pairwise manner, with the shading between genomes representative of similarity between genome pairs. Genes where a protein product was detected using mass spectrometry are highlighted in orange.

**Figure S7.** The abundance of transcripts from representative bacteriophages from the Baltic metatranscriptomic dataset.
