# Supplementary material for: Riding the wave of genomics to investigate aquatic coliphage diversity and activity
Source: Environ Microbiol. 2019 Apr 4;21(6):2112–28. doi: 10.1111/1462-2920.14590 (PMC6563131; doi:10.1111/1462-2920.14590)
Supplement: Supplementary file 1 — Appendix S1: Supporting Information [file EMI-21-2112-s001.docx]

**Supplementary Material** for:

Riding the wave of genomics to investigate aquatic coliphage diversity and activity

Slawomir Michniewski^1^, Tamsin Redgwell^1^, Aurelija Grigonyte^1^, Branko Rihtman^1^, Maria Aguilo-Ferretjans^1^, Joseph Christie-Oleza^1^, Eleanor Jameson^1^, David J. Scanlan^1^ & Andrew D. Millard^2^.

**Supplementary Tables**

**Table S1.** Core-genes, ANI and genes used for phylogenetic analysis of phages within the genus *RB69virus.* All phages were re-annotated to ensure consistent gene calling. ANI was calculated using autoANI. See attached excel file.

**Table S2.** Core-genes, ANI, and genes used for phylogenetic analysis of phages within the genus *T5*virus. All phages were re-annotated to ensure consistent gene calling. ANI was calculated using autoANI. See attached excel file.

**Table S3.** Core-genes, ANI, and genes used for phylogenetic analysis of phages within the subfamily *Tunavirinae.* ANI was calculated using autoANI. See attached excel file.

**Table S4:** Genome properties of bacteriophages: vB_Eco_mar004NP2, SWAN, vB_Eco_mar002J1, vB_Eco_mar002J2, vB_Eco_mar003J3, vB_Eco_mar005P1, vB_Eco_mar005P2, vB_Eco_mar005P3vB_Eco_mar005P4, vB_Eco_mar005P5 and vB_Eco_mar005P6. See attached excel file.

**Table S5a.** Proteomic analysis of phages vB_Eco_swan01, vB_Eco_mar005P1, vB_Eco_mar002J2, vB_Eco_mar003J3 and vB_Eco_mar004NP2.

| **Phage** |  | **Locus Tag of Detected Protein** |  | **Product** | **Locus Tags of Homologues** |
| --- | --- | --- | --- | --- | --- |
| vB_Eco_mar003J3 |  | MAR003J3_00081 |  | phage tail fibers |  |
| vB_Eco_mar003J3 |  | MAR003J3_00086 |  | phage tail length tape-measure protein | MAR004NP2_00155 |
| vB_Eco_mar003J3 |  | MAR003J3_00090 |  | major tail protein | MAR004NP2_00151 |
| vB_Eco_mar003J3 |  | MAR003J3_00094 |  | major head protein precursor | MAR004NP2_00147 |
| vB_Eco_mar003J3 |  | MAR003J3_00095 |  | putative prohead protease | MAR004NP2_00146 |
| vB_Eco_mar003J3 |  | MAR003J3_00096 |  | putative tail protein | MAR004NP2_00145 |
| vB_Eco_mar003J3 |  | MAR003J3_00097 |  | portal protein | MAR004NP2_00144 |
| vB_Eco_mar004NP2 |  | MAR004NP2_00144 |  | portal protein | MAR003J3_00097 |
| vB_Eco_mar004NP2 |  | MAR004NP2_00145 |  | putative tail protein | MAR003J3_00096 |
| vB_Eco_mar004NP2 |  | MAR004NP2_00146 |  | putative prohead protease | MAR003J3_00095 |
| vB_Eco_mar004NP2 |  | MAR004NP2_00147 |  | major head protein precursor | MAR003J3_00094 |
| vB_Eco_mar004NP2 |  | MAR004NP2_00151 |  | major tail protein | MAR003J3_00090 |
| vB_Eco_mar004NP2 |  | MAR004NP2_00155 |  | pore-forming tail tip protein | MAR003J3_00086 |
| vB_Eco_mar004NP2 |  | MAR004NP2_00157 |  | tail protein Pb3 | MAR003J3_00084 |
| vB_Eco_mar004NP2 |  | MAR004NP2_00160 |  | putative tail fiber protein |  |
| vB_Eco_mar005P1 |  | MAR005P1_00047 |  | tail sheath |  |
| vB_Eco_mar005P1 |  | MAR005P1_00051 |  | prohead core protein |  |
| vB_Eco_mar005P1 |  | MAR005P1_00054 |  | major capsid protein |  |
| vB_Eco_mar005P1 |  | MAR005P1_00076 |  | ADP-ribosyltransferase |  |
| vB_Eco_mar005P1 |  | MAR005P1_00015 |  | hypothetical protein |  |
| vB_Eco_swan01 |  | SWAN_00017 |  | tail tape-measure protein | MAR001J1_00002, MAR002J2_00028, LT841304_00017, LT961732_00067 |
| vB_Eco_swan01 |  | SWAN_00019 |  | major tail protein | MAR001J1_00004, MAR002J2_00030, LT841304_00019, LT961732_00065 |
| vB_Eco_swan01 |  | SWAN_00025 |  | putative major capsid protein | LT841304_00025, LT961732_00059, MG241338_00049 |
| vB_Eco_swan01 |  | SWAN_00026 |  | hypothetical protein | MAR001J1_00011, MAR002J2_00037, LT841304_00026 |
| vB_Eco_swan01 |  | SWAN_00027 |  | hypothetical protein | MAR001J1_00012, MAR002J2_00038, LT841304_00027 |

**Table 5b** Peptides Detected in of phages vB_Eco_swan01, vB_Eco_mar005P1, vB_Eco_mar002J2, vB_Eco_mar003J3 and vB_Eco_mar004NP2. See attached excel file.

**Table S6.** Host range of coliphages vB_Eco_swan01, vB_Eco_mar005P1, vB_Eco_mar002J2, vB_Eco_mar003J3 and vB_Eco_mar004NP2 against Enterobacteriaceae hosts. Infected hosts are marked with a black box and those that are not infected with a -.

| Host bacterial strains | Phage Isolate | | | | |
| --- | --- | --- | --- | --- | --- |
|  | vB_Eco_mar002J2 | vB_Eco_mar003J3 | vB_Eco_mar004NP2 | vB_Eco_mar005P1 | vB_Eco_swan01 |
| *Escherichia coli* MG1655 (K12) | 1 | 1 | 1 |  | 1 |
| *Escherichia coli* GD45 | - | - | - | - | - |
| *Escherichia coli* GU48 | - | - | 1 | - | - |
| *Escherichia coli* T3-21 | - | - | - | - | - |
| *Escherichia coli* SFR-11 | - | - | - | - | - |
| *Escherichia coli* D22 | 1 | 1 | 1 | - | 1 |
| *Escherichia coli* N43 | 1 | 1 | 1 | 1 | 1 |
| *Escherichia coli* EV36 | 1 | 1 | 1 | 1 | 1 |
| *Escherichia coli* 170713 | - | - | - | - | - |
| *Escherichia coli* 170972 | - | - | - | - | - |
| *Klebsiella varicola* DSM 15968 | - | - | - | - | 1 |
| *Klebsiella oxytoca* DSM 5175 | - | - | - | - | - |
| *Klebsiella oxytoca* DSM 25736 | - | - | - | - | - |
| *Klebsiella quasipneumoniae* DSM28211 | - | - | - | - | - |
| *Klebsiella michiganensis* DSM 25444 | - | - | - | - | - |
| *Klebsiella pneumoniae pneumoniae* DSM30104 | - | - | - | - | - |
| *Klebsiella pneumoniae* isolate 170723 | - | - | - | - | - |
| *Klebsiella oxytoca* isolate 170748 | 1 | - | 1 | - | - |
| *Klebsiella pneumoniae* isolate 170820 | - | 1 | - | - | - |
| Klebsiella oxytoca isolate isolate 170821 | - | - | - | - | - |
| *Klebsiella pneumoniae* isolate 170958 | 1 | 1 | 1 | - | - |
| *Klebsiella pneumoniae* isolate 171167 | 1 | 1 | - | - | - |
| *Klebsiella oxytoca* isolate 171266 | - | - | - | - | - |
| *Klebsiella pneumoniae* isolate 170304 | - | - | - | - | - |
| *Salmonella typhimurium* | - | - | 1 | - | - |

**Supplementary Figure Legends**

**Figure S1.** Phylogenetic analysis of phages within the genus *RB69virus.* The tree is based on the nucleotide sequence of the major capsid protein (*g23*), using a TIM2+F+R5 model of evolution, with 1000 bootstrap replicates using IQTREE (Nguyen *et al.*, 2015). The phages included in the tree are vB_MmoM_MP1 (acc:KX078569), PS2 (acc:KJ025957), phiR1-RT (acc:HE956709), vB_YenM_TG1 (acc:KP202158), JSE (acc:EU863408), *Aeromonas* phage 25 (acc:DQ529280), 44RR2 (acc:AY375531), 44RR2.8t.2 (acc:KY290948) , *Aeromonas* phage 31.2 (acc:KY290951), *Aeromonas* phage 31 (acc:AY962392), Riv-10 (acc:KY290957) , L9-6 (acc:KY290956), SW69-9 (acc:KY290958) , Acj9 (acc:HM004124), Ac42 (acc:HM032710), Acj61 (acc:GU911519), Merlin (acc:KT001915), Moon (acc:KM236240), CF1 (acc:MG250484), STML-198 (acc:JX181825), Melville (acc:MF957259), vB_SnwM_CGG4-1 (acc:KU867307), KP1 (acc:MG751100), PKO111 (acc:KR269720), JD18 (acc:KT239446), vB_Kpn_F48 (acc:MG746602), PG7 (acc:KJ101592), Pet-CM3-4, (acc:LT614807), CC31 (acc:GU323318), JS10 (acc:EU863409), vB_EcoM_VR5 (acc:KP007359), SP18 (acc:GQ981382), vB_EcoM_VR20 (acc:KP007360), vB_EcoM_VR7 (acc:HM563683), vB_EcoM_VR25 (acc:KP007361), vB_EcoM_VR26 (acc:KP007362), PEi20 (acc:AP014714), PEi26 (acc:AP014715), CHI14 (acc:MF036690), CBH8 (acc:MF036691), X20 (acc:MF036692), PM2 (acc:KF835987), JS98 (acc:EF469154), IME08 (acc:HM071924), MX01 (acc:KU878969), WG01 (acc:KU878968), QL01 (acc:KT176190), Bp7 (acc:HQ829472), *E*. *coli* O157 typing phage 3 (acc:KP869101), *E*. *coli* O157 typing phage 6 (acc:KP869104), RB69 (acc:AY303349), SHSML-52-1 (acc:KX130865), vB_EcoM_PhAPEC2 (acc:KF562341), phiC120 (acc:KY703222), APCEc01 (acc:KR422352), vB_Eco_mar005P1 (acc:LR027390), Shf125875 (acc:KM407600), ST0 (acc:MF044457), HX01 (acc:JX536493), vB_EcoM_JS09 (acc:KF582788), HP3 (acc:KY608965), RB59 (acc:KM607003), RB55 (acc:KM607002), T4 strain wild (acc:KJ477684), T4 (acc:AF158101), slur07 (acc:LN881732), PE37 (acc:KU925172), vB_EcoM_UFV13 (acc:KU867876), T4T (acc:HM137666), T4 strain GT7 (acc:KJ477686), T4 strain 147 (acc:KJ477685), ime09 (acc:JN202312), vB_CroM_CrRp10 (acc:MG775043), Shfl2 (acc:HM035025), RB14 (acc:FJ839692), vB_EcoM_112 (acc:KJ668714), RB51 (acc:FJ839693), RB68 (acc:KM607004), vB_EcoM_ACG-C40 (acc:JN986846), SHFML-26 (acc:KX130862), EC121 (acc:MF001359), RB32 (acc:DQ904452), RB33 (acc:KM607001), pSs-1 (acc:KM501444), SH7 (acc:KX828711), PST (acc:KF208315), SG1 (acc:MF001354), Sf22 (acc:MF158045), EC04 (acc:MF001360), slur03 (acc:LN881728), slur14 (acc:LN881736), slur08 (acc:LN881733), Sf21 (acc:MF327007), SHBML-50-1 (acc:KX130864), KPN1 (acc:KX452694), KPN5 (acc:KX452698), SF25 (acc:MF327009), Sf24 (acc:MF327008), ECML-134 (acc:JX128259), HY01 (acc:KF925357), PEC04 (acc:KR233165), UFV-AREG1 (acc:KX009778), RB3 (acc:KM606994), RB6 (acc:KM606996), RB9 (acc:KM606998), RB10 (acc:KM606999), RB7 (acc:KM606997), RB5 (acc:KM606995), RB27 (acc:KM607000), wV7 (acc:HM997020), *E*. *coli* O157 typing phage 7 (acc:KP869105), AR1 (acc:AP011113), Sf23 (acc:MF158046), SHFML-11 (acc:KX130861), HY03 (acc:KR269718), vB_EcoM-fFiEco06 (acc:MG781190), vB_EcoM-fFiEco01 (acc:MG781191), YUEEL01 (acc:KY290975), CF2 (acc:KY608967), phiD1 (acc:HE956711), slur02 (acc:LN881726), slur13 (acc:LN881737), slur11 (acc:LN881734), slur04 (acc:LN881729). Phages in the genus ***RB69virus*** are coloured in blue

**Figure S2.** Phylogenetic analysis of phages within the genus *T5virus.* The phylogenetic tree is based on the nucleotide sequence of the gene encoding DNA polymerase, using a TIM2+F+R3 model of evolution, with 1000 bootstrap replicates using IQTREE (Nguyen *et al.*, 2015). The phages included in the tree are HTVC010P (acc:NC_020481), phiR201 (acc:HE956708), saus132 (acc:MF431737), poul149 (acc:MF431738), saus176N (acc:MF431741), chee158 (acc:MF431739), chee130_1 (acc:MF431736), cott162 (acc:MF431740), vB_Eco_mar003J3 (acc:LR027389), Stitch (acc:KM236244), EPS7 (acc:CP000917), BSP22A (acc:KY787212), SH9 (acc:MF001363), 100268_sal2 (acc:KU927497), 118970_sal2 (acc:KX017521), LVR16A (acc:MF681663), APCEc03 (acc:KR422353), slur09 (acc:LN887948), SP3 (acc:MG387042), bV_EcoS_AKFV33 (acc:HQ665011), SPC35 (acc:HQ406778), SP01 (acc:KY114934), SSP1 (acc:KY963424), vB_Eco_mar004NP2 (acc:LR027384), phiLLS (acc:KY677846), Shivani (acc:KP143763), SHSML-45 (acc:KX130863), OSYSP (acc:MF402939), T5, st0 del mutant (acc:AY692264), T5 (acc:AY543070), T5,ATCC 11303-B5 (acc:AY587007), DT57C (acc:KM979354), DT571/2 (acc:KM979355), vB_EcoS_FFH1 (acc:KJ190157), pork27 (acc:MF431731), poul124 (acc:MF431735), saus47N (acc:MF431733), saus111K (acc:MF431734), chee24 (acc:MF431730), pork29 (acc:MF431732).

**Figure S3.** Phylogenetic analysis of phages within the subfamily *Tunavirinae.* The tree is based on the nucleotide sequence of the terminase gene, using a TIM2+F+R3 model of evolution, with 1000 bootstrap replicates using IQTREE (Nguyen *et al.*, 2015). The phages included in the tree are phiEt88 (acc:FQ482085), JMPW2 (acc:KU194205), T1 (acc:NC_005833) , JMPW1 (acc:KU194206), Shfl1 (acc:NC_015456), ADB-2 (acc:NC_019725), pSf-2 (acc:NC_026010), Esp2949-1 (acc:NC_019509), vB_Eco_mar001J1 (acc:LR027388), vB_Eco_mar002J2 (acc:LR027385), pSf-1 (acc:KC710998), vB_Eco_swan01 (acc:LT841304), SECphi27 (acc:LT961732), SP126 (acc:KC139513), TLS (acc:NC_009540), YSP2 (acc:MG241338), Stevie (acc:NC_027350), PKP126 (acc:NC_031053), F20 (acc:JN672684), KLPN1 (acc:KR262148), 1513 (acc:KP658157), KP36 (acc:NC_029099), MezzoGao (acc:MF612072), Sushi (acc:KT001920), GML-KpCol1 (acc:MG552615), Rtp (acc:NC_007603), vB_Eco_ACG-M12 (acc:NC_019404), vB_EcoS_Rogue1 (acc:NC_019718), phiJLA23 (acc:KC333879), C119 (acc:KT825490), e4/1c (acc:NC_024210), vB_EcoS_AKS96 (acc:NC_024789), vB_EcoS_AHP42 (acc:NC_024793), bV_EcoS_AHP24 (acc:KF771236), vB_EcoS_AHS24 (acc:NC_024784).

**Figure S4**. Genomic alignment of phages vB_Eco_mar004NP2 and vB_Eco_mar003J3. Genomes were re-ordered from the gene encoding the terminase large subunit to allow ease of comparison. Genomes were compared with EasyFig (Sullivan *et al.*, 2011) using blastn (minimum length 100 e-value 0.001). Genes detected by mass spectrometry are shaded in yellow.

**Figure S5**. Comparative genomic analysis of the genus *Rb69virus.* All phages were compared to the type phage RB69 (accession: AY303349) using BRIG (Alikhan *et al.*, 2011). From the inside out, each ring represents a blastn similarity (e-value 0.001) to phage RB69. The darker the shading within each ring, the higher the similarity. The outer two rings mark the genes and annotation as extracted from the Genbank file (AY303349).

**Figure S6.** Comparative analysis of the proposed genus *psFunavirus.* All genomes were re-ordered with the gene encoding the terminase subunit as a starting point. Genomes were compared with blastn (minimum length -100, e-value -0.001) using EasyFig. Genomes were compared in a pairwise manner, with the shading between genomes representative of similarity between genome pairs. Genes where a protein product was detected using mass spectrometry are highlighted in orange.

**Figure S7.** The abundance of transcripts from representative bacteriophages from the Baltic metatranscriptomic dataset.

**Supplementary Material and Methods**

Complete bacteriophage genomes were downloaded from Genbank using esearch and efetch

esearch -db nucleotide -query "gbdiv_PHG[prop]" | efilter -query "1417:700000 [SLEN] " |efetch -format gb > phagedb.gbk

Phages were then filtered to remove incomplete phage genomes based on manual inspection of the Genbank files. These included accessions

CTU76612|KF59418[456789]|KF59419[01234]|EF136589|EF69468[45678]|EF69469[01234]|DQ221100|AF158601|AF034975|DQ198146|EU794053|EU794050|AY780364|AF151091|AB012112|X72793|JF906059|JF906060|JF906059|KC13963[246]|KC13956[123456]|KC13954[34]|KC13952[678]|AF031901|AF071201|KX50113[456789]|MPU46938|KT31959[789]|KJ09402[56]|KJ668716|AF195901|AY846870|PT4G55G43|CTU76612|KY197768|MF498774|M14782|DQ198146|Y10775|X91069|EU794053|EU794051|EU794050|EU794054|EU794052|AF071201|DQ221100|AF034975|DQ221100|AJ413274|NC_004302|U38906|AF031901|X17255|M10160|EF694684|EF694685|EF694686|EF694687|EF694688|EF694689|EF694690|EF694691|EF694693|EF694694|X72793|AB012112|MF001357|U76612|KR131711|AY780364|AJ292531|V01127|KJ094025|KJ094026|KJ094027|KJ094028|KX501134|KX501135|KX501136|KX501137|KX501138|KX501139|KC139528|KC139526|KC139527|KC139544|KC139543|KC139563|KC139566|KC139561|KC139560|KC139562|KC139564|KC139652|KC139559|KC139634|KC139632|KC139636|KC139524|KC139523|KC139650|KC139649|KC139667|KC139680|KC139640|KC139638|KC139513|KY775452|KY775453|MF001364|JX233783|KF594191|KF594184|KF594185|KF594186|KF594187|KF594192|KF594193|KF594188|KF594194|KF594189|KF594190|EF136589|DQ364602|KT337344|KT337345|KT337346|KT337347|KT337348|KT337349|KT337351|KT337353|KT337367|KT337369|KT337372|NC_003157|AY050245|AF348736|AF158601|KM389296|KM389298|KM389300|KM389302|KM389304|KM389305|KM389386|KM389387|KM389388|KM389389|KM389390|KM389391|KM389392|KY608967|KY608965|KX452694|KM389360|KM389434|KM389435|KM389252|KM389306|KM389287|KM389288|KF879861|KF879862|KF879863|KM389324|KM389325|KM389459|KM389216|KM389237|KM389238|KM389401|KM389402|KM389326|KM389225|KM389226|KM389460|KM389229|KM389233|KM389234|KM389242|KM389461|KM389462|KM389336|KM389337|KM389266|KM389267|KM389212|KM389247|KM389463|KM389464|JQ067086|JQ067088|JQ067083|JQ067090|JQ067091|KM389373|KM389374|KM389219|KM389220|KM389278|KF879864|KM389214|KM389215|KM389222|KM389280|KM389416|KM389417|KM389348|KF723224|KF664568|KF664567|KF664566|GQ485652|GQ485648|GQ485647|GQ485644|GQ485649|GQ466609|GQ466608|GQ466612|GQ466610|GQ485650|GQ485651|GQ485646|GQ485645|HQ906665|HQ906666|AB161975

The complete script for downloading and creating files is available here http://s3.climb.ac.uk/Sinfo/Download_bacteriophage_genomes.pl

**Bioinformatics analysis of the Baltic Sea virome**

The abundance of representative bacteriophages in the Baltic Virome (DNA)**.** Genome coverage was calculated using BBMap with the following options

bbmap.sh ref=rep_phage.fna in=$*read1* in2=$*read2* covstats=$cov minid=$minid outm=$mapped

$*read1 –*forward read file

$read2 = reverse read file – when 2 files were present

$covstats – coverage of the genome

$mapped – the –mapped output reads in fastq file

The Baltic Virome fasta data available from iMicrobe under project code CAM_P_0001109

The coverage data presented was calculated by the covstats function within BBMap.

Reads from the metatranscriptomics dataset were sequentially downloaded using fasterq-dump from the short read archive. The accessions of files used are: SRR3745322, SRR3745332, SRR3745334, SRR3745335, SRR3745337, SRR3745339, SRR3745340, SRR3745341, SRR3745342, SRR3745343, SRR3745344, SRR3745347, SRR3745356, SRR3746048, SRR3747289, SRR3747315, SRR3747324, SRR3747325, SRR3747326, SRR3747327, SRR3747328, SRR3747329, SRR3747330, SRR3747331, SRR3747359, SRR3747360, SRR3747361, SRR3747362, SRR3747363, SRR3747364, SRR3747368, SRR3747400, SRR3747401, SRR3747402, SRR3747403, SRR3747404, SRR3747405, SRR3747412, SRR3747413, SRR3747414, SRR3747415, SRR3747416, SRR3747417, SRR3747418, SRR3747419, SRR3747420, SRR3747762, SRR3747763, SRR3747764, SRR3747765, SRR3747775, SRR3747777, SRR3747797, SRR3747799, SRR3747800, SRR3747801, SRR3747802, SRR3747803, SRR3747804, SRR3747805, SRR3747806, SRR3747807, SRR3747808, SRR3747809, SRR3747810, SRR3747823, SRR3747824.

Reads were again stringently mapped to a single file that contained all representative genomes using BBMap with the settings ‘minid=90, covstats, outm’ (Bushnell, 2019). The number of reads mapped to each genome was normalised for both length of the phage genome and the number of reads per sample.

The number of reads mapped to each genome was normalised for both length of the phage genome and the number of reads per sample.

$\left[ \frac{\left[ Number of reads{mapped}/{Reference} genome size \left( kb \right) \right]}{\left[ Number of reads in database \right]} \right]x 1 000,000$

To give the number of reads mapped per kb of phage genome per million reads in the database. This data was plotted for each sampling site as extracted from (Zeigler Allen *et al.*, 2017). Reads that mapped to the new bacteriophage genomes were then subsequently used in a BLAST search against the nr database.

**References**

Alikhan, N.F., Petty, N.K., Ben Zakour, N.L., and Beatson, S.A. (2011) BLAST Ring Image Generator (BRIG): Simple prokaryote genome comparisons. *BMC Genomics*.

Bushnell, B. (2019) BBTools. *http://sourceforge.net/projects/bbmap/sourceforge.net/projects/bbmap/*.

Nguyen, L., Schmidt, H.A., von Haeseler, A., and Minh, B.Q. (2015) IQ-TREE: A fast and effective stochastic algorithm for estimating maximum-likelihood phylogenies. *Mol. Biol. Evol.* **32**: 268–274.

Sullivan, M.J., Petty, N.K., and Beatson, S.A. (2011) Easyfig: A genome comparison visualizer. *Bioinformatics*.

Zeigler Allen, L., McCrow, J.P., Ininbergs, K., Dupont, C.L., Badger, J.H., Hoffman, J.M., et al. (2017) The Baltic Sea virome: diversity and transcriptional activity of DNA and RNA viruses. *mSystems* **2**: e00125-16.
