# Supplementary figures and images for: Riding the wave of genomics to investigate aquatic coliphage diversity and activity
Source: Environ Microbiol. 2019 Apr 4;21(6):2112–28. doi: 10.1111/1462-2920.14590 (PMC6563131; doi:10.1111/1462-2920.14590)

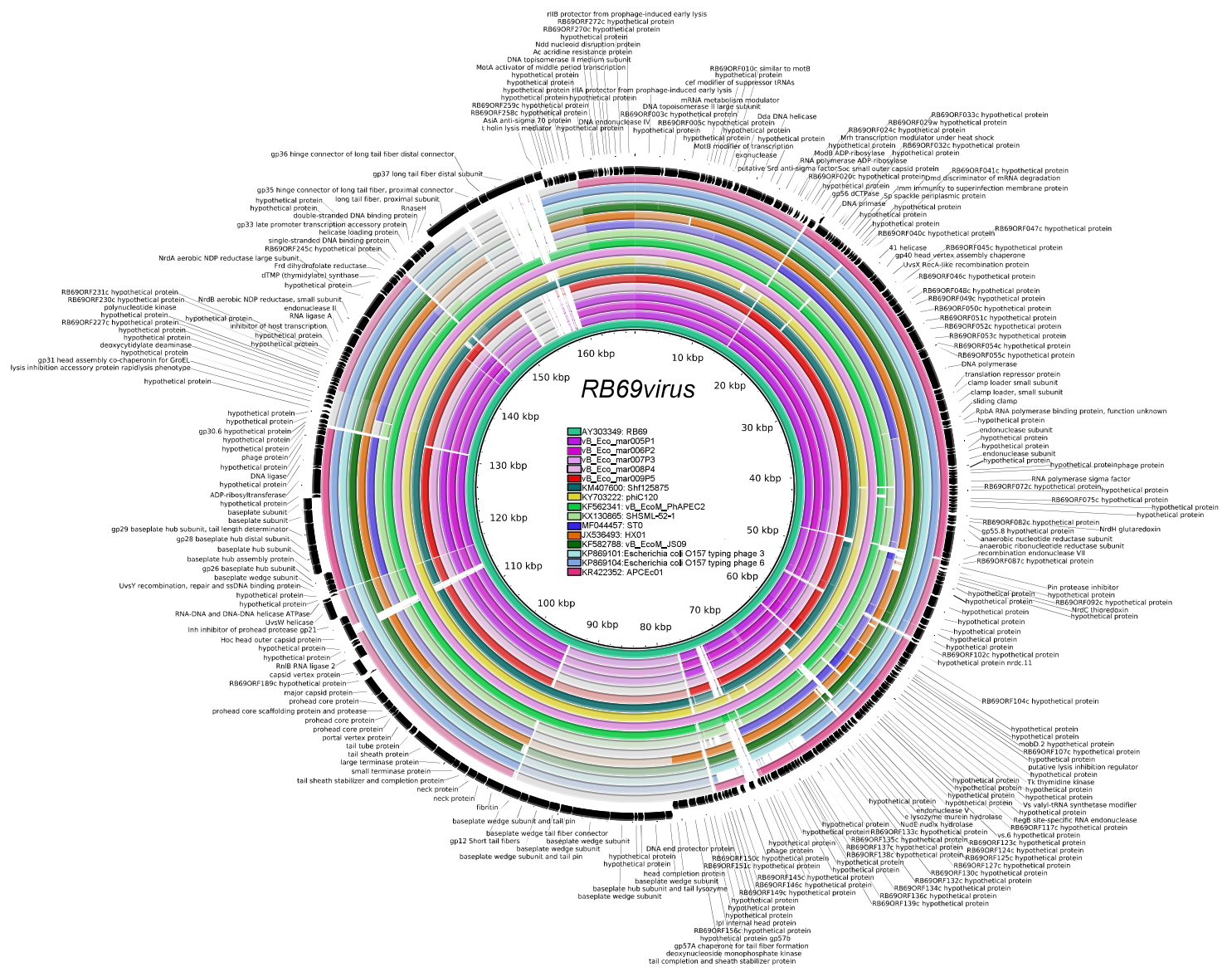

Supplement: Supplementary file 11 — Figure S5. Comparative genomic analysis of the genus Rb69virus. All phages were compared to the type phage RB69 (accession: AY303349) using BRIG (Alikhan et al., 2011). From the inside out, each ring represents a blastn similarity (e‐value 0.001) to phage RB69. The darker the shading within each ring, the higher the similarity. The outer two rings mark the genes and annotation as extracted from the Genbank file (AY303349). [file EMI-21-2112-s011.pdf]

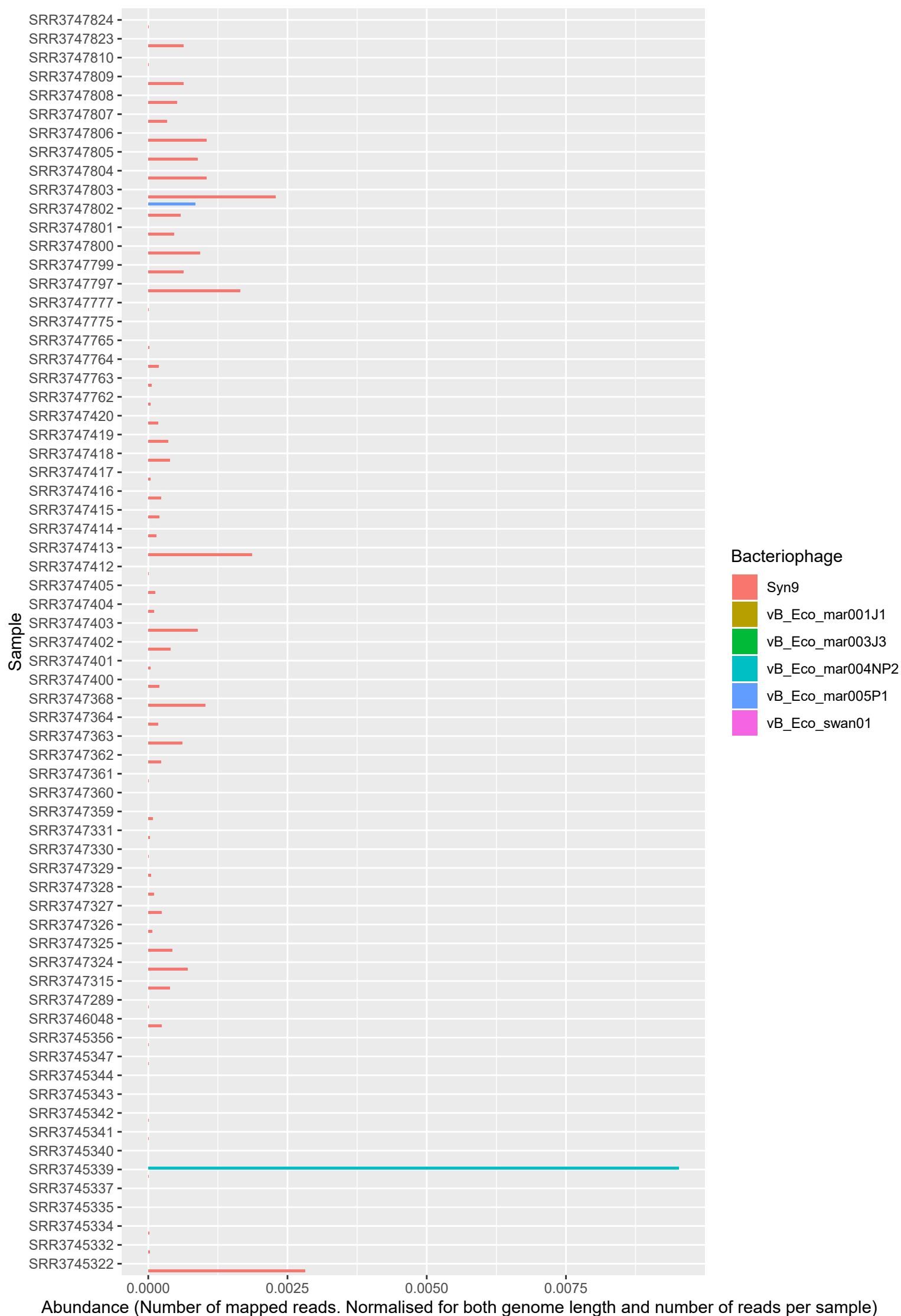

Supplement: Supplementary file 13 — Figure S7. The abundance of transcripts from representative bacteriophages from the Baltic metatranscriptomic dataset. [file EMI-21-2112-s013.pdf]
